# Dietary silicate: a biogenic strategy to enhance growth, gut health, and nutrient utilization in finishing pigs through low-carbon diets

**DOI:** 10.1186/s40813-026-00494-5

**Published:** 2026-02-21

**Authors:** Wei Han Zhao, Seung Jin Yun, In Ho Kim

**Affiliations:** 1 Department of Animal Biotechnology, Dankook University, Cheonan, 31187 Korea; 2 Smart Animal Bio Institute, Dankook University, Cheonan, 31187 Korea

**Keywords:** Silicate, Growth performance, Gas emissions, Meat quality, Fecal microbiota, Low-carbon diets

## Abstract

**Background:**

Low-carbon feeding is an effective strategy to reduce nutrient losses and environmental emissions in swine production. High-protein (HP) diets increase nitrogen (N) excretion and ammonia (NH_3_) emissions, whereas low-protein (LP) diets improve N utilization but may impair growth performance. Silicate (SIL) has been reported to enhance digestion and reduce harmful gas emissions; however, its efficacy under different dietary protein levels remains unclear. Therefore, this study evaluated the effects of SIL supplementation in HP and LP diets to provide evidence for efficient and low-carbon pig production.

**Methods:**

A 10-wk feeding trial was conducted using 200 pigs [Duroc × (Landrace × Yorkshire)] with an initial body weight of 55.40 ± 3.36 kg. Pigs were randomly assigned to four dietary treatments in a randomized complete block design, with 10 replicates per treatment and 5 pigs per pen (2 gilts and 3 barrows). The experiment followed a 2 × 2 factorial arrangement, consisting of two dietary protein levels: HP and LP diets, with 2% lower crude protein (CP), and two levels of SIL supplementation (0 or 0.1%).

**Results:**

The results demonstrated that dietary supplementation with 0.1% SIL significantly improved growth performance, as indicated by increased average daily gain (ADG) and reduced feed conversion ratio (FCR) (*P < 0.05*). SIL supplementation also enhanced the digestibility of dry matter (DM), N, and energy (E), while reducing moisture digestibility (*P* < 0.05). In addition, SIL significantly decreased fecal emissions of NH₃, hydrogen sulfide (H₂S), and carbon dioxide (CO₂) and improved fecal consistency scores at wk 10 (*P* < 0.05), indicating its potential to mitigate environmental emissions. Regarding physiological responses, SIL supplementation increased white blood cell (WBC) counts and blood glucose concentrations (*P* < 0.05 and *P* < 0.01, respectively), while reducing serum creatinine and cortisol levels (*P* < 0.05), suggesting improved metabolic status and reduced physiological stress. In terms of meat quality, SIL enhanced muscle water-holding capacity (WHC), reduced drip loss, and improved tissue cohesiveness and elasticity (*P* < 0.05). Moreover, SIL supplementation increased the proportion of unsaturated fatty acids and improved the ratio of saturated to unsaturated fatty acids, indicating a favorable modification of lipid composition. Dietary protein level also exerted significant main effects. LP diets reduced CO₂ emissions and blood urea nitrogen (BUN) concentrations (*P* < 0.05), reflecting a lower N metabolic burden and reduced carbon emissions. No significant interaction between dietary protein level and SIL supplementation was observed for the measured parameters. Fecal microbiota analysis showed that SIL supplementation was associated with alterations in gut microbial composition, characterized by an increased relative abundance of beneficial taxa, including Lactobacillus and members of the Lactobacillaceae family. These microbial shifts may contribute to improved intestinal health and more favorable fermentation characteristics, thereby supporting the observed reductions in harmful gas emissions.

**Conclusion:**

Dietary inclusion of 0.1% SIL represents an effective nutritional strategy for enhancing growth performance and nutrient utilization in finishing pigs, while supporting a low-carbon feeding model. By improving gut health and reducing environmental emissions, SIL demonstrates strong potential as a functional feed additive for promoting both productivity and environmental sustainability in pig production.

## Introduction

In recent years, approximately 25–30% of global greenhouse gas (GHG) emissions have originated from the food system, with livestock production being one of the major contributors [1]. As the degree of intensification in livestock farming continues to increase, swine production accounts for a substantial proportion of the total emissions within the food chain [2]. Therefore, optimizing pig production systems, reducing production costs, and implementing low-carbon dietary models are key approaches to achieving GHG mitigation and sustainable livestock development [3]. To address the growing environmental challenges, many recent studies have focused on adjusting dietary structures such as reducing the proportion of high carbon footprint ingredients like soybean meal, enhancing the utilization of locally sourced feed materials, and supplementing functional additives to improve feed efficiency which can effectively reduce the carbon emissions per unit of pork produced [4]. Lowering CP concentration in pig diets is an effective strategy to reduce N excretion and greenhouse gas emissions [5]; however, it may lead to declines in growth performance and feed efficiency [6]. Therefore, current research has focused on identifying nutritional strategies [7], enzymes [8] and probiotics [9] combinations that can mitigate these negative effects while maintaining animal productivity and environmental sustainability. The dietary CP level is a key factor in pig nutrition: where HP diets can promote rapid growth, excessive protein intake increases the N content of feces and urine, resulting in elevated NH₃ emissions, N loss, and water eutrophication [10]. Therefore, reducing dietary protein levels while supplementing crystalline essential amino acids has become a feasible approach to lower N excretion and feed costs [11]. For example, studies have shown that a 2–4% reduction in dietary protein levels, combined with balanced ratios of essential amino acids, significantly reduced N excretion without adversely affecting growth performance or carcass traits [12]. However, when CP levels are further reduced, nutrient digestion and metabolic efficiency may be compromised, and changes in lipid metabolism have been observed, including increased intramuscular fat deposition and altered fatty acid composition [13]. These limitations highlight the necessity of integrating functional feed additives to support digestive function and metabolic balance under LP, low-carbon feeding systems.

SIL minerals, owing to their unique physicochemical properties, have recently gained attention as novel functional feed additives [14]. Natural or modified SIL (such as zeolite and diatomite) possess strong adsorption, ion-exchange, and surface-reactivity capacities, effectively binding ammonium ions and amino compounds, thereby slowing urea N release and reducing NH_3_ emissions [15]. SIL-based feed additives can also prevent NH_3_ and heavy-metal toxicity, making them useful for adsorbing mycotoxins and mitigating feed contamination; thus, they have been widely applied as mycotoxin binders in livestock production [16]. In pigs, studies have found that supplementation of 0.1% SIL in the basal diet significantly increased body weight, ADG, and gain-to-feed ratio during the finishing period, while markedly improving CP digestibility, increased white blood cell and lymphocyte counts, enhancing immune status, reducing fecal Escherichia coli abundance, and significantly decreasing NH_3_ emissions. These results indicate that SIL may enhance mucosal immunity, modulate gut microbiota, and improve urea N utilization, thereby optimizing nutrient conversion and host health [17]. Mechanistically, SIL may exert their biological effects by modulating the intestinal physicochemical environment, potentially through the adsorption of alkaline nitrogenous metabolites such as NH_3_, thereby lowering intestinal pH. As a central regulator of host nutrient metabolism, the gut microbiota plays a critical role in N utilization, fecal gas production, and lipid metabolism [18]. A mildly acidified intestinal environment favors the selective proliferation of beneficial microorganisms, especially lactic acid bacteria, while suppressing NH_3_-producing and potentially pathogenic bacteria [19]. Previous studies support this mechanism; for example, piglets fed acid-loaded diatomite diets exhibited significantly reduced intestinal pH, elevated lactic acid concentrations, and a marked increase in *Lactobacillus* abundance [20]. The enrichment of lactic acid bacteria can shift microbial fermentation patterns toward increased production of short-chain fatty acids (SCFAs), such as acetate and butyrate. These metabolites not only serve as major energy (E) sources for intestinal epithelial cells but also play important roles in maintaining intestinal barrier integrity, regulating lipid metabolism, and improving N utilization efficiency. In addition, lactic acid bacteria can inhibit pathogen growth through competitive exclusion and organic acid production, while enhancing tight junction integrity and mucosal immune function. The lactic acid they produce also acts as a key substrate for microbial cross-feeding, promoting SCFAs synthesis by other microbial taxa and thereby reducing the accumulation of substrates for proteolytic fermentation and NH_3_ production [21]. Collectively, these microbiota-mediated processes provide a plausible mechanistic explanation for the reduced N losses and harmful gas emissions observed following SIL supplementation. Studies involving SIL or similar minerals (such as synthetic zeolites) have demonstrated that these additives reduce fecal *E. coli* and other harmful bacteria while promoting beneficial microbes and immunoglobulin production, thereby decreasing emissions of NH_3_ and H_2_S [22]. Compared with previous studies that primarily focused on single indicators such as growth performance or NH_3_ emissions, the present study extends existing knowledge by comprehensively evaluating SIL supplementation under different dietary CP levels within a low-carbon framework. In summary, SIL supplementation may reduce NH_3_ emissions and optimize feed nutrient utilization by altering the gut microbial (particularly by increasing the proportion of lactobacilli) community and chemical environment, thereby suppressing the activity of NH_3_-producing bacteria.

Although several studies have explored the effects of SIL or zeolite-type minerals in pigs, comprehensive assessments of these additives under different dietary protein levels on growth performance, environmental emissions, meat quality, and gut microbiota remain limited. Moreover, limited information is available regarding whether SIL supplementation can function synergistically with LP diets to achieve both productivity maintenance and carbon-emission reduction. Therefore, the present study aimed to evaluate the multifaceted effects of SIL supplementation in diets with varying CP levels on finishing pigs, including growth performance, nutrient digestibility, blood biochemical parameters, fecal characteristics, harmful gas emissions, meat physicochemical properties, and fatty acid profiles. Particular attention was given to the structural and functional shifts in the intestinal microbiota and their associations with environmental and meat-quality outcomes. By integrating LP nutrition with functional mineral supplementation, this study seeks to provide a low-carbon feeding strategy supported by multi-index biological evidence, thereby offering a scientific rationale for the application of SIL minerals in sustainable pig production systems.

## Materials and methods

### Source of SIL

A sodium SIL, produced by Myoungjeonbio Co., Ltd. (183-7, Doha 3-gil, Munbaek-myeon, Jincheon-gun, Chungcheongbuk-do, Republic of Korea, 27868), was added to the experimental diets. The product was manufactured using a patented process for producing soluble silicate compositions (Patent No. 10-2064226) and is further protected under Patent No. 10-2305003. Soluble SIL was produced using high-purity quartz (SiO₂; ≥95%) as the primary raw material. The manufacturing process is as follows:

1. Quartz mineral powder is ground into fine particles of 200–300 mesh size.
2. Impurities are removed using screening devices (screens, drum separators, etc.).
3. 100 parts by weight of quartz mineral powder are mixed with 30–40 parts by weight of sodium carbonate (Na₂CO₃) and 10–20 parts by weight of phosphate compounds (H_3_PO₄).
4. The mixed quartz minerals are introduced into an electric furnace or blast furnace and heated at a high temperature of 2000–2200℃ for 8–12 h to undergo melting.
5. After melting, the material is slowly cooled in the air to form solidified SIL.
6. The solidified SIL is crushed into small particles and subjected to a drying process.
7. A solubility test is conducted to confirm whether the manufactured SIL (Na₂SiO₃·10 H₂O) is completely soluble in water.
8. The content of insoluble SIL is controlled to be no more than 5% to ensure high purity.
9. The chemical composition is analyzed by X-ray diffraction (XRD) and inductively coupled plasma mass spectrometry (ICP-MS), and the mineral composition and ion-exchange capacity are evaluated.
10. The final product is ground to a particle size of 200–300 mesh, making it suitable for use as an animal feed additive.
11. If necessary, 40–50 parts by weight of shell powder and 20–30 parts by weight of liquid sulfur are mixed.
12. The SIL is then added to 500–600 parts by weight of a phospholipid mixture to produce the animal feed additive.

## Study design

**Fig. 1:**
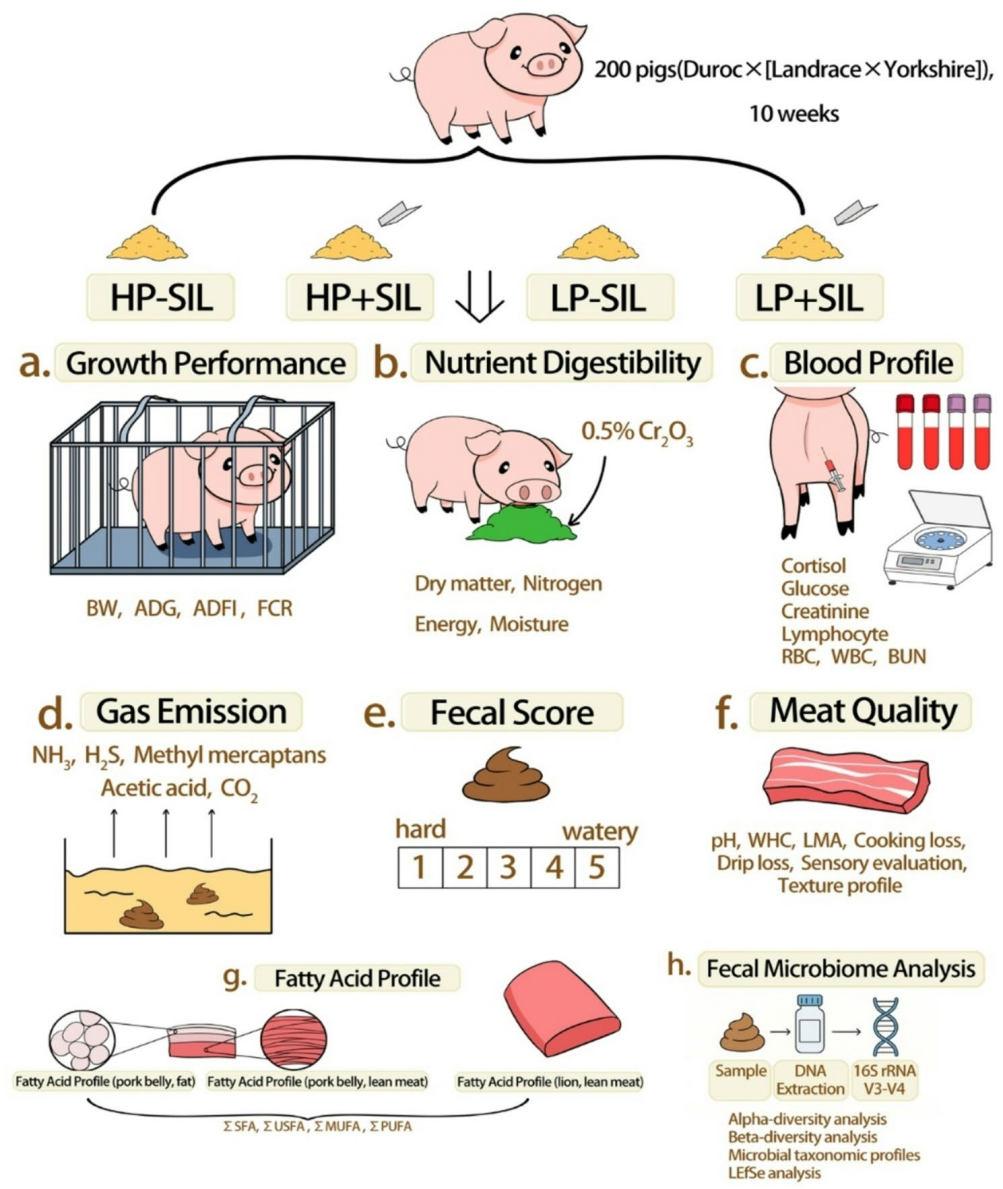
Experimental flow design diagram. HP = high protein; LP = low protein; SIL = silicate; BW = body weight; ADG = average daily gain; ADFI = average daily feed intake; FCR = feed conversion ratio; RBC = red blood cell; WBC = white blood cell; BUN = blood urea nitrogen; WHC = water holding capacity; LMA = longissimus muscle area

A total of 200 crossbred weaned pigs [Duroc × (Landrace × Yorkshire)] with an initial average body weight of 55.40 ± 3.36 kg were assigned to four treatment groups using a randomized block design based on body weight. The feeding trial lasted for 10 wk. Each treatment consisted of 10 replicate pens, with five pigs per pen (including two gilts and three barrows). Four dietary treatments were established: (1) HP − SIL; (2) HP + SIL; (3) LP − SIL; and (4) LP + SIL. A schematic diagram of the experimental design is shown in Fig. 1 Diets were formulated to meet or exceed the nutrient requirements of weaned pigs as recommended by the National Research Council [23], with details provided in Table 1. All pigs were housed in a temperature-controlled facility with plastic slatted floors and a mechanical ventilation system. The chemical composition of the basal diets was determined according to [24] procedures, including DM (AOAC 930.15), N (AOAC 984.13), CP (calculated as 6.25 × N), and crude fat (AOAC 920.39). Ca (AOAC 927.02) and P (AOAC 965.17) were also measured. All analyses were performed in six replicates to ensure data accuracy. Each pen measured 1.8 × 1.8 m and was equipped with a stainless-steel automatic feeder and nipple drinkers to allow ad libitum access to feed and water. Artificial lighting was maintained for 12 h per d, and the ambient temperature was kept at 22.5 °C. Routine inspections were conducted daily at 08:00, 12:00, 16:00, and 20:00 to ensure adequate feed and water availability and to monitor the health status of the pigs.

**Table 1:** Composition of finishing pig diets^1^

Item	Experimental diet	
	Basal diet	Basal diet − 2% CP
Ingredients (g/kg)		
Corn	710.2	759.2
Soybean meal	130.6	76.5
DDGS	100.0	100.0
Tallow	26.5	27.4
MDCP	11.0	12.7
Limestone	7.3	6.4
Salt	3.0	3.0
Methionine (99%)	0.5	0.8
Lysine (78%)	5.0	6.9
Threonine (99%)	1.1	2.0
Tryptophan (99%)	0.5	0.8
Mineral mix^2^	2.0	2.0
Vitamin mix^3^	2.0	2.0
Choline (25%)	0.3	0.3
Total	1000.0	1000.0
Analyzed value		
Dry matter (g/kg)	890.4	890.6
Crude protein, (g/kg)	148.4	127.7
Ca, (g/kg)	6.12	5.97
P, (g/kg)	5.84	5.92
ME, (kacl/kg) Calculated	3286	3314
Crude fat, (g/kg)	61.4	63.5

^1^ Abbreviation: DDGS, distillers dried grains solubles; MDCP, mono dicalcium phosphate; ME, metabolizable energy; LYS, lysine; MET, methionine^2^ Provided per kg diet: Fe, 115 mg as ferrous sulfate; Cu, 70 mg as copper sulfate; Mn, 20 mg as manganese oxide; Zn, 60 mg as zinc oxide; I, 0.5 mg as potassium iodide; and Se, 0.3 mg as sodium selenite^3^ Provided per kilograms of diet: vitamin A, 13,000 IU; vitamin D_3_, 1,700 IU; vitamin E, 60 IU; vitamin K_3_, 5 mg; vitamin B_1_, 4.2 mg; vitamin B_2_, 19 mg; vitamin B_6_, 6.7 mg; vitamin B_12_, 0.05 mg; biotin, 0.34 mg; folic acid, 2.1 mg; niacin, 55 mg; D-calcium pantothenate, 45 mg

### Growth performance

During the 10-wk experimental period, the body weight (BW) of each pig was individually measured and recorded at the start of the trial, as well as at wk 5 and 10, using a GL-6000 portable electronic scale. Based on these measurements, the ADG was calculated for each treatment group. In addition, daily feed intake and feed refusals were recorded to determine the average daily feed intake (ADFI), which was subsequently used to calculate the FCR.

$$\:\mathrm{A}\mathrm{D}\mathrm{G}\:\left(\mathrm{g}/\mathrm{d}\right)=\frac{\mathrm{F}\mathrm{i}\mathrm{n}\mathrm{a}\mathrm{l}\:\mathrm{B}\mathrm{W}-\:\mathrm{I}\mathrm{n}\mathrm{i}\mathrm{t}\mathrm{i}\mathrm{a}\mathrm{l}\:\mathrm{B}\mathrm{W}\:\left(\mathrm{g}\right)}{\mathrm{E}\mathrm{x}\mathrm{p}\mathrm{e}\mathrm{r}\mathrm{i}\mathrm{m}\mathrm{e}\mathrm{n}\mathrm{t}\mathrm{a}\mathrm{l}\:\mathrm{d}\mathrm{a}\mathrm{y}\mathrm{s}\:}$$

$$\:\mathrm{A}\mathrm{D}\mathrm{F}\mathrm{I}\left(\mathrm{g}/\mathrm{d}\right)=\frac{\mathrm{T}\mathrm{o}\mathrm{t}\mathrm{a}\mathrm{l}\:\mathrm{F}\mathrm{e}\mathrm{e}\mathrm{d}\:\mathrm{I}\mathrm{n}\mathrm{t}\mathrm{a}\mathrm{k}\mathrm{e}\:\left(\mathrm{g}\right)}{\mathrm{E}\mathrm{x}\mathrm{p}\mathrm{e}\mathrm{r}\mathrm{i}\mathrm{m}\mathrm{e}\mathrm{n}\mathrm{t}\mathrm{a}\mathrm{l}\:\mathrm{d}\mathrm{a}\mathrm{y}\mathrm{s}\:}$$

$$\:\mathrm{F}\mathrm{C}\mathrm{R}=\frac{\mathrm{F}\mathrm{e}\mathrm{e}\mathrm{d}\:\mathrm{I}\mathrm{n}\mathrm{t}\mathrm{a}\mathrm{k}\mathrm{e}\:}{\mathrm{W}\mathrm{e}\mathrm{i}\mathrm{g}\mathrm{h}\mathrm{t}\:\mathrm{G}\mathrm{a}\mathrm{i}\mathrm{n}\:}$$

### Nutrient digestibility

To determine the apparent total tract digestibility (ATTD), 0.50% chromium oxide (Cr₂O₃) was added to the diets during the last 7 d of the experiment. Feed samples were collected in sterile bags and stored. Fecal samples were collected via rectal massage from one sow and one barrow per pen at the same time each morning for three consecutive days, and samples were then pooled by pen. Feed and fecal samples were stored at − 20 °C until analysis of DM, N, E, and chromium content. Prior to chemical analysis, all samples were dried at 60 °C for 72 h, finely ground, and passed through a 1 mm screen to ensure homogeneity. Nutrient digestibility and chromium absorption were determined according to the methods of [24] and the procedures described by [25]. Chromium content was measured using a UV spectrophotometer (Optizen POP, Seoul, Korea); E was determined with a bomb calorimeter (Parr 6100, Moline, IL, USA); and N was analyzed with a Kjeldahl N analyzer (Kjeltec 2300, Foss Tecator AB, Hillerød, Denmark). Fecal moisture content was calculated using the following formula:

$$\begin{aligned}&\mathrm{M}\mathrm{o}\mathrm{i}\mathrm{s}\mathrm{t}\mathrm{u}\mathrm{r}\mathrm{e}\:\mathrm{c}\mathrm{o}\mathrm{n}\mathrm{t}\mathrm{e}\mathrm{n}\mathrm{t}\:\left(\mathrm{\%}\right)\cr& \quad=\frac{\mathrm{F}\mathrm{r}\mathrm{e}\mathrm{s}\mathrm{h}\:\mathrm{w}\mathrm{e}\mathrm{i}\mathrm{g}\mathrm{h}\mathrm{t}\:-\:\mathrm{D}\mathrm{r}\mathrm{y}\:\mathrm{w}\mathrm{e}\mathrm{i}\mathrm{g}\mathrm{h}\mathrm{t}}{\mathrm{F}\mathrm{r}\mathrm{e}\mathrm{s}\mathrm{h}\:\mathrm{w}\mathrm{e}\mathrm{i}\mathrm{g}\mathrm{h}\mathrm{t}}\:\times\:\:100\end{aligned}$$

ATTD was calculated using the following formula:

$$\begin{aligned}&\mathrm{A}\mathrm{T}\mathrm{T}\mathrm{D}\:\left(\mathrm{\%}\right)\cr &=\:[1\:-\:\{({N}_{f}\times\:\:{\mathrm{C}}_{d})\:/\:({\mathrm{N}}_{d}\:\times\:\:{\mathrm{C}}_{f}\}]\cr &\times\:\:100\end{aligned}$$

where Nf represents nutrient concentration in feces (% DM), Cd is chromium concentration in the diet (% DM), Nd is nutrient concentration in the diet (% DM), and Cf is chromium concentration in feces (% DM).

### Gas emission

At the end of wk 10, fresh fecal samples (300 g) were collected simultaneously from each treatment group. The samples were placed into 2,600-mL airtight plastic containers, ensuring that approximately two-thirds of the container volume was left empty to allow for gas accumulation. Each container was immediately sealed to prevent gas leakage and incubated at 25 °C for 7 days under dark and undisturbed conditions.

After fermentation, the containers were gently shaken to homogenize the headspace gas before measurement. Gas concentrations of NH₃, H₂S, CO₂, methyl mercaptan, and acetic acid were determined using a calibrated multi-gas detector (Multi-RAE Lite, Model PGM-6208, RAE Systems, San Jose, CA, USA). Prior to measurements, the detector was zero-calibrated in fresh air according to the manufacturer’s guidelines. Gas detection was conducted by inserting the device’s sampling probe through a sealed port in the container lid to avoid gas escape and ensure stable readings.

### Blood profile

At the end of wk 10, two pigs (one barrow and one gilt) were randomly selected from each pen at the same time, and jugular blood samples were collected using the standing restraint method by trained personnel. Before sampling, the puncture site was disinfected with 70% ethanol, and blood was collected using a 10-mL disposable syringe.

For hematological analysis, 2 mL of blood was transferred into tripotassium EDTA (K₃EDTA) anticoagulant tubes (Becton Dickinson, USA). The samples were gently inverted 8–10 times immediately after collection to prevent clotting. Hematological parameters, including white blood cell (WBC), red blood cell (RBC), and lymphocyte counts, were analyzed within 2 h using an automated hematology analyzer (ADVIA^®^ 120, Bayer, USA).

For serum biochemical analysis, 5 mL of blood was collected into coagulation tubes and allowed to clot at room temperature for 30 min. The samples were then centrifuged at 3000 × g for 15 min, and serum levels of BUN, creatinine, glucose, and cortisol were measured using an automated biochemical analyzer (HITACHI 747, Tokyo, Japan).

### Fecal score

At wk 5 and 10 of the trial, two trained technicians conducted fecal scoring for each pen over seven consecutive days, and the average score was calculated. Scoring was performed at the same time each morning to minimize the effects of diurnal variation, following the method described by [26], and the average score was calculated. A 5-point scoring system was used as follows: 1 = hard, dry, small pellet-like feces forming relatively firm clumps; 2 = firm, well-formed feces, solid but slightly soft; 3 = soft, formed, and moist feces that still maintain their shape; 4 = loose, unformed feces that take the shape of the container; 5 = watery, liquid-like feces that can be poured directly. Scoring was performed at the pen level, based on observations of the individual pigs and the overall fecal characteristics within each pen.

### Meat quality

After 10 wk of feeding, eight pigs (4 barrows and 4 gilts) were randomly selected from each treatment group and transported to a nearby slaughterhouse, where they were fasted and rested for 6 h. The pigs were stunned by electrical shock and then exsanguinated. The carcasses were subsequently transferred to a cutting room maintained at 0–4 °C. After 45 min of postmortem aging, a 1 kg sample of the left longissimus dorsi muscle was collected, stored at 4 °C for 24 h, and then prepared for cutting and analysis. Muscle pH was measured using a pH meter (Istek Model 77p, Korea), calibrated at 25 °C with a pH 7.00 buffer solution. WHC was determined according to [27], with the area measured using a planimeter (X-plan, Ushikata 360d, Ushikata, Japan) and expressed as the ratio of the meat surface area to the water area. The cross-sectional area of the longissimus dorsi was traced onto OHP film and measured using an area meter (MT-10 S, MT Precision, Tokyo, Japan). Meat color was measured using a colorimeter (Chromameter CR-410, Minolta, Japan). Each sample was measured twice, and the mean value was recorded. Calibration was performed using a standard color tile with the following parameters: L* = 89.2, a* = 0.921, b* = 0.783 (D-65 light source, 2° observer angle). Cooking loss, defined as water loss during heating due to contraction of myofibrillar proteins and connective tissue, was determined by shaping approximately 2 g of each sample to a fixed size, placing it in a polyethylene bag, and heating in a 75 °C water bath for 30 min. After cooling for 30 min at room temperature, samples were reweighed to calculate cooking loss. Drip loss, representing weight reduction caused by water loss from myofibrillar contraction over time, was measured on two 2.0 × 2.0 cm pork loin samples collected from each carcass, placed in drip loss tubes, stored at 4 °C, and weighed on days 1, 3, 5, and 7.

Sensory evaluation was conducted by a panel of 10 trained male and female staff. Vacuum-packaged samples were thawed at 4 °C for 2 h, cut into 15 mm thick slices, and allowed to stand for 30 min for color assessment based on intramuscular fat content and color standards. Samples were scored for color, marbling, and firmness on a 1–5 scale.

After thawing at 4 °C for 12 h, samples of the longissimus dorsi and longissimus thoracis muscles were cut into 50 mm thick slices (*n* = 25 per treatment), trimmed of all fat and connective tissue, and cut along the muscle fiber direction. Texture Profile Analysis (TPA) was performed using a texture analyzer (Lloyd TA1, USA) to simulate oral mastication and evaluate the physical properties of the samples. Two-centimeter-thick samples were placed under a cylindrical probe, with a pre-test speed of 3.0 mm/s and a test speed of 1.0 mm/s. The probe was compressed into the sample to a set deformation, returned, and after a 2 s interval, a second compression was applied. Force values were recorded every 0.01 s to generate force–time curves [28] from which hardness (maximum compression force, indicating firmness), cohesiveness (ability to maintain original structure, reflecting internal bonding strength), adhesiveness (force required to separate the sample from the probe, indicating stickiness), and springiness (distance of recovery after compression, reflecting elasticity) were determined.

### Fatty acid profile

Crude fat determination: Ten grams of lean meat sample were mixed with 5 g of anhydrous sodium sulfate, dried in an oven at 100 °C, and ground. The sample was placed into a cylindrical filter paper, and crude fat was extracted using a fat extractor (ST 243 soxtexTM) and a constant-weight flask. Fifty milliliters of anhydrous ether were added, boiled at 70 °C for 20 min, continuously distilled and extracted for 50 min, and the solvent was recovered for 10 min. After drying to remove the residual solvent, the flask was cooled and weighed to calculate crude fat content. The extracted crude fat was used for fatty acid composition analysis.

Fatty acid analysis method: A 25 mg sample (pork belly, fat; pork belly, lean meat; loin, lean meat) was completely dissolved in 1.5 mL of 0.5 N methanolic sodium hydroxide solution, heated at 100 °C for 5 min, and cooled. The mixture was then combined with 2 mL of 14% BF₃ solution, heated for 30 min, cooled to 30–40 °C, followed by the addition of 1 mL of iso-octane and 5 mL of saturated sodium chloride solution. The supernatant was collected for fatty acid analysis. Crude fat (%) calculation formula:

$$\begin{aligned}&\mathrm{C}\mathrm{r}\mathrm{u}\mathrm{d}\mathrm{e}\:\mathrm{f}\mathrm{a}\mathrm{t}\:\left(\mathrm{\%}\right)\cr & \quad=\:\frac{\begin{aligned}& \mathrm{W}\mathrm{e}\mathrm{i}\mathrm{g}\mathrm{h}\mathrm{t}\:\mathrm{o}\mathrm{f}\:\mathrm{e}\mathrm{x}\mathrm{t}\mathrm{r}\mathrm{a}\mathrm{c}\mathrm{t}\mathrm{i}\mathrm{o}\mathrm{n}\cr &\mathrm{f}\mathrm{l}\mathrm{a}\mathrm{s}\mathrm{k}\:\mathrm{a}\mathrm{f}\mathrm{t}\mathrm{e}\mathrm{r}\:\mathrm{e}\mathrm{x}\mathrm{t}\mathrm{r}\mathrm{a}\mathrm{c}\mathrm{t}\mathrm{i}\mathrm{o}\mathrm{n}\cr & -\:\mathrm{W}\mathrm{e}\mathrm{i}\mathrm{g}\mathrm{h}\mathrm{t}\:\mathrm{o}\mathrm{f}\:\mathrm{e}\mathrm{x}\mathrm{t}\mathrm{r}\mathrm{a}\mathrm{c}\mathrm{t}\mathrm{i}\mathrm{o}\mathrm{n}\cr &\mathrm{f}\mathrm{l}\mathrm{a}\mathrm{s}\mathrm{k}\:\mathrm{b}\mathrm{e}\mathrm{f}\mathrm{o}\mathrm{r}\mathrm{e}\:\mathrm{e}\mathrm{x}\mathrm{t}\mathrm{r}\mathrm{a}\mathrm{c}\mathrm{t}\mathrm{i}\mathrm{o}\mathrm{n}\end {aligned}}{\mathrm{S}\mathrm{a}\mathrm{m}\mathrm{p}\mathrm{l}\mathrm{e}\:\mathrm{w}\mathrm{e}\mathrm{i}\mathrm{g}\mathrm{h}\mathrm{t}}\: \times\:\:100\end{aligned}$$

Individual fatty acid content (%) formula:

$$\begin{aligned}&\mathrm{I}\mathrm{n}\mathrm{d}\mathrm{i}\mathrm{v}\mathrm{i}\mathrm{d}\mathrm{u}\mathrm{a}\mathrm{l}\:\mathrm{f}\mathrm{a}\mathrm{t}\mathrm{t}\mathrm{y}\:\mathrm{a}\mathrm{c}\mathrm{i}\mathrm{d}\:\left(\mathrm{\%}\right)\cr & \quad=\:\frac{{\mathrm{W}\mathrm{F}\mathrm{A}\mathrm{M}\mathrm{E}}_{i}}{\mathrm{T}\mathrm{o}\mathrm{t}\mathrm{a}\mathrm{l}\:\mathrm{p}\mathrm{e}\mathrm{a}\mathrm{k}\:\mathrm{a}\mathrm{r}\mathrm{e}\mathrm{a}}\:\times\:\:100\end{aligned}$$

### 16S rRNA sampling

Fecal DNA was extracted using the QIAamp PowerFecal Pro DNA Kit (Qiagen, Hilden, Germany) following the manufacturer’s instructions. The concentration and purity of genomic DNA were measured using a UV spectrophotometer (Molecular Devices, CA, USA). The V3–V4 hypervariable region of the 16S rRNA gene was amplified and sequenced on the Illumina MiSeq platform (Illumina, CA, USA), with sequencing services provided by CJ BioScience, Inc. (Seoul, Korea). Raw sequencing data were processed using the QIIME2 pipeline [29]. Primer and adapter sequences were removed using the “cutadapt” plugin [30], and quality filtering, denoising, and feature table construction were performed using the DADA2 algorithm [31]. Within-sample microbial diversity was assessed using alpha diversity indices, including Observed features, Chao1, Shannon, Simpson, and Pielou’s evenness, while between-sample differences were evaluated via principal coordinate analysis (PCoA) based on Bray Curtis dissimilarity and UniFrac distances. Taxonomic annotation and relative abundance analyses were conducted at the phylum, family, and genus levels. To identify microbial taxa exhibiting significant differences at the phylum, family, and genus levels, Linear Discriminant Analysis Effect Size (LEfSe) was performed. Initially, the Kruskal Wallis rank-sum test was applied to identify features with statistically significant differences in abundance among groups (*p < 0.05*). Subsequently, Linear Discriminant Analysis (LDA) was conducted to estimate the effect size of each differentially abundant taxon. The results were visualized as bar plots, illustrating the LDA scores of the identified taxa.

### Statistical analysis

All experimental data were analyzed using SAS software (version 9.4; SAS Institute Inc., Cary, NC, USA). Before statistical analysis, all data were tested for normality using the Shapiro–Wilk test.

The experiment was conducted using a completely randomized 2 × 2 factorial design, and data were analyzed by two-way analysis of variance (ANOVA) to evaluate the main effects of SIL supplementation, dietary protein level, and their interaction. When no significant interaction between SIL supplementation and protein level was detected, statistical interpretation was based solely on the main effects. For analyses of growth performance, nutrient digestibility, fecal gas emissions, and fecal score, the pen was considered the experimental unit. For blood parameters, meat quality, fatty acid composition of pork, and fecal microbiota analyses, the individual pig was considered the experimental unit. Differences were considered statistically significant at *P* < 0.05, and 0.05 < *P* < 0.10 was considered a tendency.

In fecal microbiota analysis, permutational multivariate analysis of variance (PERMANOVA) based on Bray–Curtis and unweighted UniFrac distances was used to assess the effects of dietary protein level and SIL supplementation on β-diversity. Non-parametric visualization of microbial community structures was performed using GraphPad Prism version 8 (GraphPad Software Inc., CA, USA).

## Results

### Growth performance

The effects of SIL supplementation on the growth performance of finishing pigs at different dietary protein levels are presented in Table 2. As a main effect, SIL supplementation significantly increased ADG during wk 0–5, wk 5–10, and over the entire experimental period, and significantly reduced FCR during wk 5–10 and the overall period (*P < 0.05*), indicating a clear positive effect of SIL on growth performance. In contrast, the main effect of dietary protein and the interaction between protein and SIL were not significant.

**Table 2:** Effects of SIL supplementation at different protein levels in diets on growth performance of finishing pigs^1^

Items	High protein		Low protein		SEM	*P* - value		
	SIL -	SIL +	SIL -	SIL +		CP effect	SIL effect	Interaction
Body weight, kg								
Initial	55.41	55.40	55.40	55.41	1.11	0.999	0.997	0.994
Wk 5	83.78	84.73	83.16	84.3	1.33	0.691	0.433	0.943
Wk 10	117.34	119.54	114.97	118.51	1.72	0.358	0.125	0.714
Initial - Wk 5								
ADG, g	811	838	793	825	14	0.307	0.048	0.865
ADFI, g	2108	2137	2085	2125	23	0.442	0.147	0.804
FCR	2.603	2.553	2.635	2.577	0.031	0.362	0.084	0.904
Wk 5 - Wk 10								
ADG, g	959	994	909	977	19	0.128	0.020	0.445
ADFI, g	2725	2733	2638	2727	37	0.227	0.203	0.285
FCR	2.847	2.756	2.912	2.798	0.041	0.290	0.046	0.815
Overall								
ADG, g	885	916	851	901	15	0.145	0.017	0.563
ADFI, g	2417	2435	2361	2426	25	0.195	0.096	0.344
FCR	2.735	2.663	2.782	2.696	0.033	0.296	0.046	0.861

^1^Abbreviation: SIL = silicate; CP = crude protein; SEM = standard error of means; ADG = average daily gain; ADFI = average daily feed intake; FCR = feed conversion ratio;

### Nutrient digestibility

**Fig. 2:**
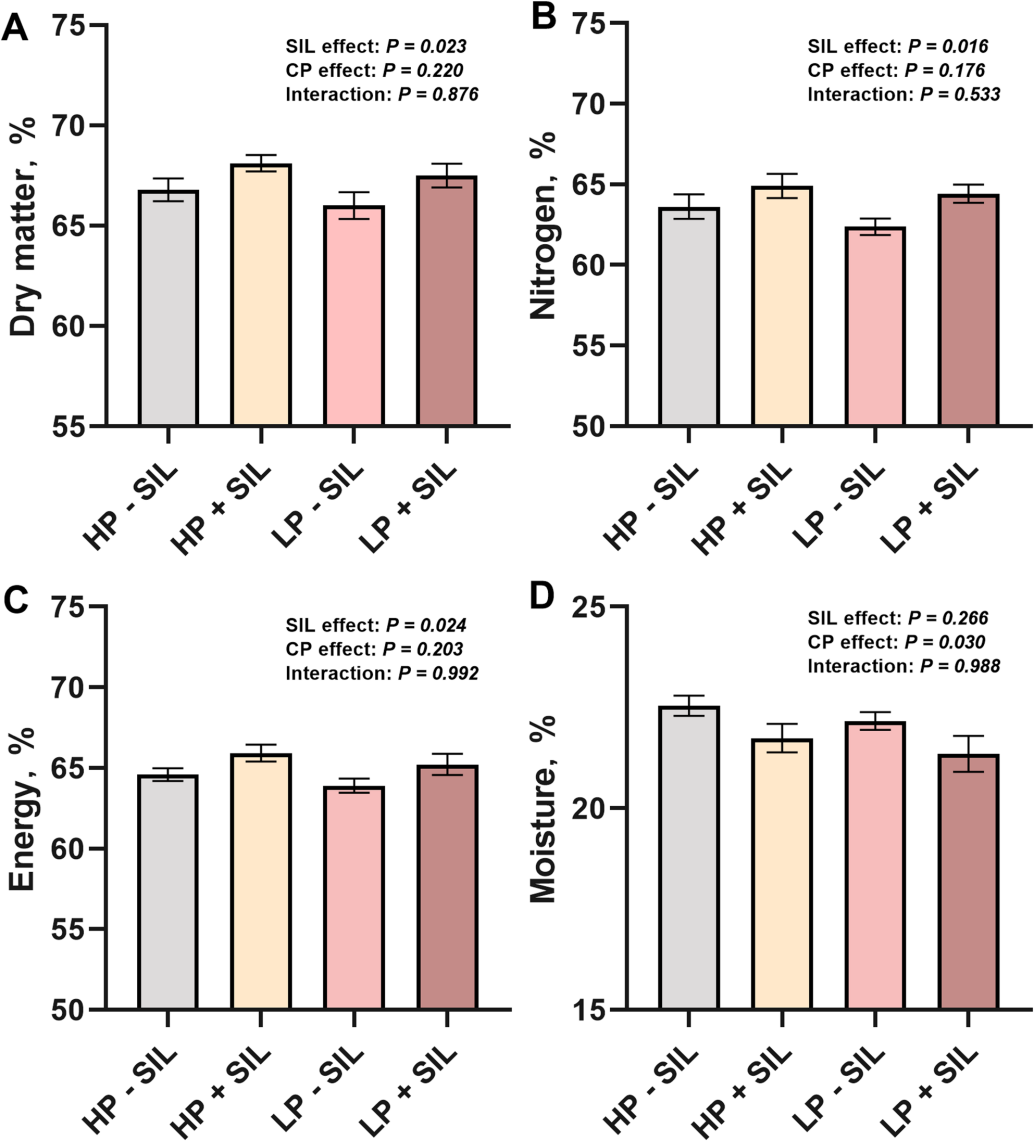
Effects of SIL supplementation at different protein levels in diets on nutrient digestibility in finishing pigs. (**A**) dry matter. (**B**) nitrogen. (**C**) energy. (**D**) moisture. Values are represented as mean ± S*EM*, *n* = 20. HP = high protein; LP = low protein; SIL = silicate

The effects of SIL supplementation on nutrient digestibility are shown in Fig. 2. As a main effect, SIL supplementation significantly improved the digestibility of DM, N, and E, and significantly reduced fecal moisture content (*P < 0.05*), indicating an overall enhancement of nutrient utilization associated with SIL supplementation. In contrast, neither the main effect of dietary protein level nor the interaction between dietary protein and SIL was significant.

### Gas emissions

**Fig. 3:**
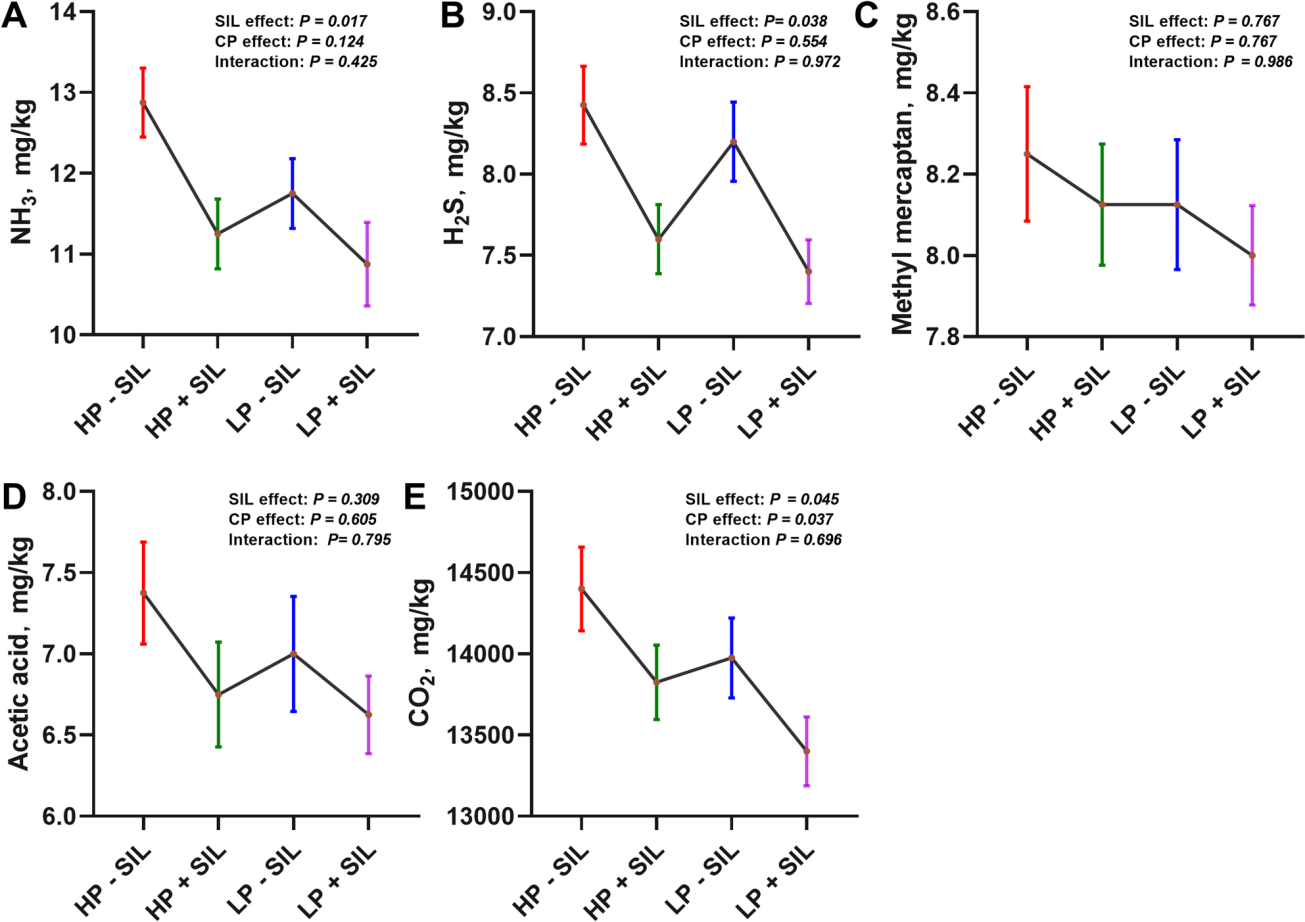
Effects of SIL supplementation at different protein levels in diets on gas emission in finishing pigs. (**A**) ammonia. (**B**) hydrogen sulfide. (**C**) methyl mercaptan. (**D**) acetic acid. (**E**) carbon dioxide. Values are represented as mean ± SEM, *n* = 10. HP = high protein; LP = low protein; SIL = silicate

The effects of SIL supplementation on gas emissions are presented in Fig. 3. As a main effect, SIL supplementation significantly reduced NH₃, H₂S, and CO₂ emissions (*P < 0.05*), indicating an overall mitigation of gaseous emissions associated with SIL supplementation. In addition, dietary protein level exerted a significant main effect on CO₂ emissions (*P < 0.05*), whereas no significant interaction between dietary protein level and SIL supplementation was observed.

### Blood parameters

**Fig. 4:**
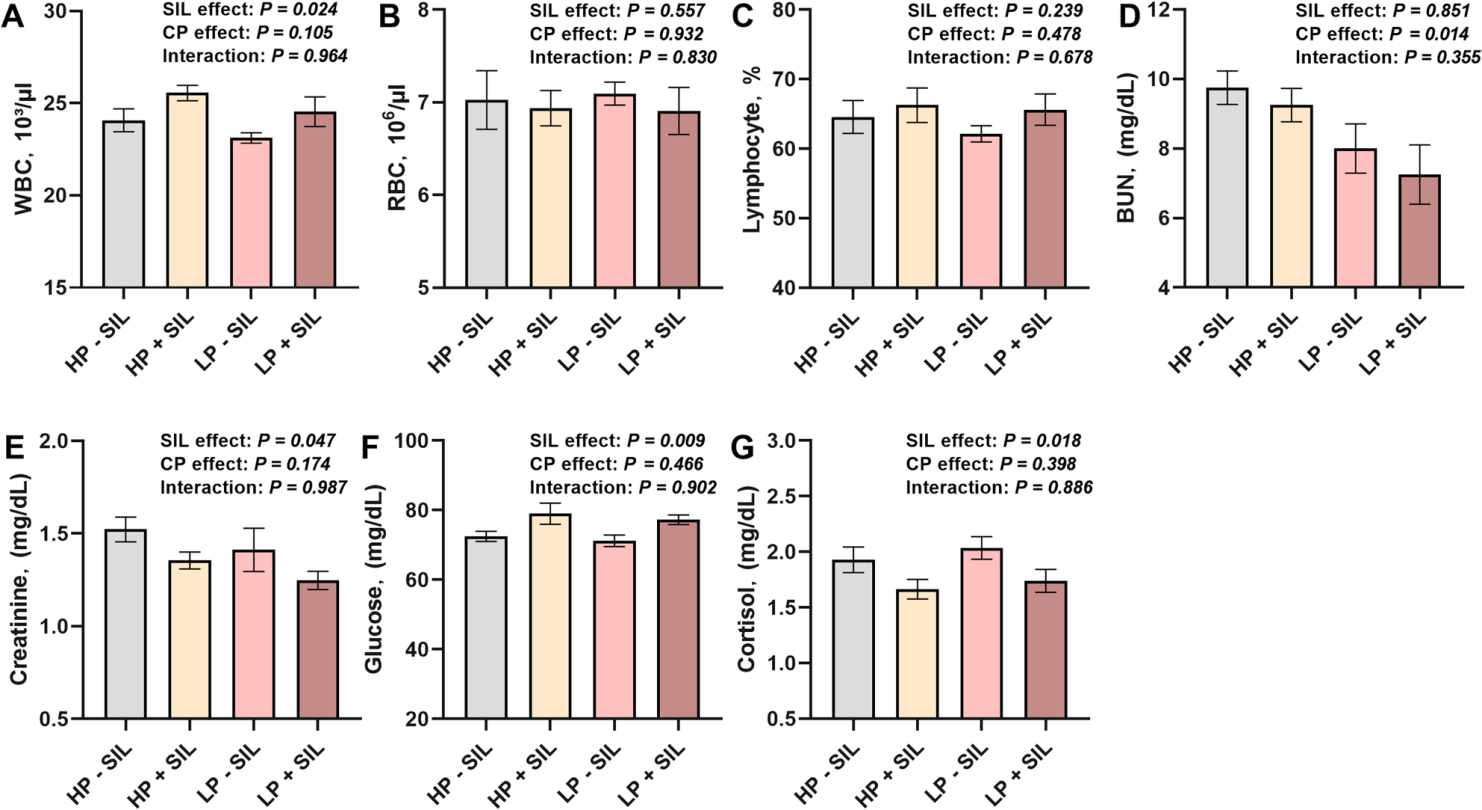
Effects of SIL supplementation at different protein levels in diets on blood profile in finishing pigs. (**A**) white blood cell. (**B**) red blood cell. (**C**) lymphocyte. (**D**) blood urea nitrogen. (**E**) creatinine. (**F**) glucose. (**G**) cortisol. Values are represented as mean ± SEM, *n* = 20. HP = high protein; LP = low protein; SIL = silicate

The effects of SIL supplementation on blood parameters are presented in Fig. 4. As a main effect, SIL supplementation significantly affected WBC count, glucose, cortisol, creatinine, and BUN concentrations (*P < 0.05*), indicating that SIL supplementation was associated with significant changes in several hematological and metabolic indicators. In addition, dietary protein level exerted a significant main effect on BUN concentration (*P < 0.05*). No significant interaction between dietary protein level and SIL supplementation was observed.

### Fecal scores

The effects of SIL supplementation on fecal scores are shown in Table 3. As a main effect, SIL supplementation significantly improved fecal scores at wk 10 (*P < 0.05*), with feces shifting from watery to more formed stools, indicating improved fecal consistency. In contrast, the main effect of dietary protein level and the interaction between dietary protein level and SIL supplementation had no significant effects on fecal scores.

**Table 3:** Effects of SIL supplementation at different protein levels in diets on fecal score in finishing pigs^1^

Items	High protein		Low protein		SEM	*P* - value		
	SIL -	SIL +	SIL -	SIL +		CP effect	SIL effect	Interaction
Fecal score^3^								
Wk 5	3.25	3.19	3.20	3.16	0.05	0.447	0.370	0.835
Wk 10	3.21	3.15	3.18	3.10	0.03	0.319	0.040	0.578

^1^Abbreviation: SIL = silicate; CP = crude protein; SEM = standard error of means

### Meat quality

The effects of SIL supplementation on meat quality are shown in Table 4. As a main effect, SIL supplementation significantly improved water-holding capacity (WHC), reduced drip loss on d 7, and increased cohesiveness and springiness (*P* < 0.05), which are closely associated with improved texture, tenderness, and overall sensory acceptability of pork. In contrast, the main effect of dietary protein level and the interaction between dietary protein level and SIL supplementation had no significant influence on these meat quality parameters.

**Table 4:** Effects of SIL supplementation at different protein levels in diets on meat quality in finishing pigs^1^

Items	High protein		Low protein		SEM	*P* - value		
	SIL -	SIL +	SIL -	SIL +		CP effect	SIL effect	Interaction
pH	5.54	5.56	5.46	5.47	0.07	0.227	0.867	0.926
Water holding capacity, %	38.30	41.83	37.56	40.45	1.42	0.461	0.040	0.822
Longissimus muscle area, cm^2^	7983.47	8065.40	7865.50	7943.33	413.56	0.777	0.850	0.996
Meat color								
L*	56.23	56.09	55.75	56.60	0.60	0.980	0.566	0.426
a*	15.96	15.53	15.74	15.34	0.74	0.792	0.583	0.988
b*	6.59	6.81	6.90	6.53	0.37	0.981	0.842	0.438
Cooking loss, %	19.38	18.96	19.89	19.45	0.64	0.448	0.513	0.988
Drip loss, %								
d1	0.98	0.93	1.06	0.99	0.15	0.611	0.693	0.942
d3	1.98	1.85	2.09	2.01	0.18	0.474	0.597	0.893
d5	4.02	3.94	4.15	4.06	0.14	0.388	0.565	0.945
d7	4.94	4.66	5.10	4.85	0.19	0.182	0.049	0.912
Sensory evaluation								
Color	3.00	3.17	3.08	3.17	0.18	0.633	0.347	1.000
Marbling	3.00	3.08	2.92	3.00	0.16	0.369	0.369	0.761
Firmness	3.17	3.25	3.08	3.17	0.13	0.539	0.539	1.000
Texture profile								
Hardness bite 1, N	146.09	149.00	133.29	137.05	8.72	0.181	0.709	0.962
Hardness bite 2, N	113.47	119.12	106.52	110.21	6.89	0.272	0.511	0.889
Cohesiveness, N	0.44	0.56	0.36	0.45	0.05	0.075	0.048	0.767
Springness, mm	0.69	0.79	0.62	0.68	0.04	0.056	0.019	0.380
Adhesiveness, Nmm	1.22	1.29	1.13	1.18	0.21	0.641	0.772	0.976

^1^Abbreviation: SIL: silicate; CP: crude protein; SEM: standard error of means

### Fatty acid profile

The effects of SIL supplementation on the fatty acid profile of pork belly (fat) are shown in Table 5. Significant SIL main effects were observed for C16:0, C18:1c, C18:3n3 (ALA), C20:0, C20:4n6, C22:2, ω-3 fatty acids, the ω-6:ω-3 ratio, ΣSFA, ΣUSFA, MUFA, and the MUFA/SFA ratio (*P < 0.001*,* < 0.001 0.033*,* 0.036*,* 0.007*,* 0.041*,* < 0.001 0.001*,* 0.006*,* 0.006*,* < 0.001*, and *< 0.001*, respectively). In contrast, neither the main effect of dietary protein level nor the interaction between dietary protein level and SIL supplementation significantly affected the fatty acid profile of pork belly fat.

**Table 5:** Effects of SIL supplementation at different protein levels in diets on fatty acid profile (pork belly, fat) in finishing pigs^1^

Items	High protein		Low protein		SEM	*P* – value		
	SIL -	SIL +	SIL -	SIL +		CP effect	SIL effect	Interaction
C4:0	0.00	0.00	0.00	0.00	-	-	-	-
C6:0	0.05	0.05	0.07	0.07	0.01	0.090	0.822	0.653
C8:0	0.03	0.03	0.03	0.03	0.003	0.961	0.718	0.749
C10:0	0.06	0.06	0.06	0.05	0.004	0.745	0.121	0.745
C11:0	0.00	0.00	0.00	0.00	-	-	-	-
C12:0	0.32	0.33	0.31	0.32	0.01	0.536	0.274	0.901
C13:0	0.00	0.00	0.00	0.00	-	-	-	-
C14:0	1.80	1.78	1.81	1.83	0.02	0.195	0.960	0.461
C14:1	0.03	0.03	0.03	0.03	0.001	0.053	0.171	0.883
C15:0	0.11	0.13	0.13	0.13	0.02	0.657	0.911	0.657
C15:1	0.00	0.00	0.00	0.00	-	-	-	-
C16:0	25.90	24.08	25.93	23.65	0.37	0.587	< 0.001	0.539
C16:1	2.78	2.59	2.63	2.40	0.14	0.237	0.167	0.877
C17:0	0.38	0.36	0.39	0.44	0.03	0.198	0.676	0.314
C17:1	0.27	0.24	0.23	0.26	0.02	0.706	0.940	0.078
C18:0	10.76	10.45	10.62	10.96	0.42	0.668	0.974	0.450
C18: 1,t	0.04	0.03	0.02	0.03	0.02	0.872	0.872	0.522
C18: 1,c	42.52	45.48	43.00	45.57	0.55	0.620	< 0.001	0.733
C18:2n6t	0.01	0.00	0.03	0.01	0.01	0.111	0.171	0.698
C18:2n6c, LA	12.05	11.32	11.88	11.19	0.59	0.800	0.250	0.977
C18:3n6	0.01	0.01	0.02	0.02	0.01	0.124	0.771	0.966
C18:3n3, ALA	0.48	0.59	0.51	0.62	0.04	0.571	0.033	0.976
C20:0	0.15	0.17	0.16	0.17	0.01	0.304	0.036	0.159
C20:1	1.01	1.03	0.89	0.97	0.01	0.126	0.318	0.604
C20:2	0.42	0.40	0.43	0.38	0.03	0.926	0.208	0.643
C20:3n6	0.06	0.06	0.05	0.06	0.004	0.180	0.410	0.781
C21:0	0.00	0.00	0.00	0.00	-	-	-	-
C20:3n3	0.11	0.11	0.08	0.11	0.01	0.133	0.436	0.204
C20:4n6	0.05	0.06	0.04	0.06	0.01	0.191	0.007	0.143
C20:5n3, EPA	0.08	0.08	0.09	0.09	0.01	0.290	0.934	0.934
C22:0	0.00	0.00	0.00	0.00	-	-	-	-
C22:1n9	0.18	0.19	0.20	0.20	0.03	0.522	0.941	0.854
C22:2	0.05	0.04	0.04	0.04	0.01	0.838	0.041	0.318
C23:0	0.05	0.05	0.04	0.05	0.00	0.781	0.070	0.410
C24:0	0.02	0.01	0.04	0.01	0.01	0.176	0.123	0.454
C22:6n3, DHA	0.15	0.18	0.12	0.16	0.03	0.490	0.324	0.877
C24:1n9	0.08	0.09	0.10	0.10	0.04	0.653	0.904	0.991
ω-3 fatty acid	0.82	0.95	0.81	0.98	0.03	0.885	0.001	0.564
ω-6 fatty acid	12.18	11.45	12.02	11.34	0.59	0.825	0.252	0.965
ω-6: ω-3	14.89	11.98	15.03	11.59	0.75	0.875	0.001	0.732
ΣSFA	39.62	37.49	39.6	37.70	0.60	0.873	0.006	0.853
ΣUSFA	60.38	62.51	60.40	62.30	0.60	0.873	0.006	0.853
ΣMUFA	46.91	49.68	47.10	49.56	0.59	0.957	0.001	0.802
ΣPUFA	13.47	12.84	13.30	12.74	0.62	0.834	0.352	0.952
MUFA/SFA	1.19	1.33	1.19	1.32	0.03	0.937	0.001	0.812
PUFA/SFA	0.34	0.34	0.34	0.34	0.02	0.846	0.949	0.949
Unknown	0.00	0.00	0.00	0.00	-	-	-	-
Total FA	100.00	100.00	100.00	100.00	-	-	-	-

^1^Abbreviation: SIL: silicate; CP: crude protein; SEM: standard error of means; LA: linoleic acid; ALA: α-Linolenic acid; EPA: eicosapentaenoic acid; DHA: docosahexaenoic acid; SFA: saturated fatty acids; USFA: unsaturated fatty acids; MUFA: monounsaturated fatty acids; PUFA: polyunsaturated fatty acids; FA: fatty acids

The effects of SIL supplementation on the fatty acid profile of pork belly (lean meat) are presented in Table 6. Significant SIL main effects were detected for C10:0, C16:0, C17:0, C18:2n6c (LA), C18:3n3 (ALA), C20:3n6, C20:3n3, C20:4n6, C22:6n3 (DHA), ω-3 fatty acids, ω-6 fatty acids, and the ω-6:ω-3 ratio (*P = 0.036*,* 0.001*,* < 0.001 < 0.001 < 0.001*,* < 0.001 < 0.001*,* < 0.001 0.035*,* < 0.001*,* < 0.001*, and *< 0.001*, respectively). No significant main effects of dietary protein level or protein × SIL interactions were observed for these parameters.

**Table 6:** Effects of SIL supplementation at different protein levels in diets on fatty acid profile (pork belly, lean meat) in finishing pigs^1^

Items	High protein		Low protein		SEM	*P* - value		
	SIL -	SIL +	SIL -	SIL +		CP effect	SIL effect	Interaction
Crude fat	46.50	42.69	47.53	46.77	2.56	0.339	0.390	0.564
C4:0	0.00	0.00	0.00	0.00	-	-	-	-
C6:0	0.12	0.12	0.13	0.14	0.01	0.255	0.339	0.561
C8:0	0.02	0.03	0.03	0.04	0.01	0.300	0.274	0.936
C10:0	0.07	0.07	0.07	0.07	0.001	0.963	0.036	0.107
C11:0	0.00	0.00	0.00	0.00	-	-	-	-
C12:0	0.31	0.31	0.30	0.31	0.01	0.583	0.366	0.583
C13:0	0.00	0.00	0.00	0.00	-	-	-	-
C14:0	1.92	1.90	1.95	1.89	0.03	0.769	0.183	0.533
C14:1	0.04	0.04	0.04	0.04	0.01	0.617	0.796	0.837
C15:0	0.35	0.33	0.34	0.37	0.03	0.660	0.791	0.300
C15:1	0.00	0.00	0.00	0.00	-	-	-	-
C16:0	23.13	22.46	23.49	21.61	0.31	0.445	0.001	0.073
C16:1	2.66	2.67	2.66	2.68	0.01	0.704	0.114	0.734
C17:0	0.31	0.35	0.32	0.37	0.01	0.058	< 0.001	0.852
C17:1	0.20	0.22	0.21	0.23	0.02	0.391	0.243	0.950
C18:0	12.81	12.49	12.07	13.64	0.54	0.711	0.272	0.109
C18: 1,t	0.04	0.04	0.04	0.04	0.003	0.431	0.271	0.503
C18: 1,c	47.07	47.92	47.31	47.56	0.50	0.912	0.311	0.575
C18:2n6t	0.04	0.04	0.04	0.04	0.005	0.545	0.744	0.824
C18:2n6c, LA	7.66	7.43	7.67	7.44	0.02	0.680	< 0.001	0.918
C18:3n6	0.03	0.03	0.03	0.03	0.003	0.710	0.476	0.631
C18:3n3, ALA	0.33	0.45	0.35	0.46	0.02	0.547	< 0.0.001	0.762
C20:0	0.19	0.18	0.20	0.19	0.01	0.407	0.517	0.926
C20:1	1.25	1.33	1.27	1.31	0.05	0.922	0.234	0.660
C20:2	0.29	0.30	0.29	0.32	0.03	0.719	0.551	0.661
C20:3n6	0.04	0.04	0.04	0.03	0.002	0.411	< 0.001	0.101
C21:0	0.00	0.00	0.00	0.00	-	-	-	-
C20:3n3	0.07	0.09	0.07	0.08	0.004	0.748	< 0.001	0.343
C20:4n6	0.03	0.04	0.03	0.04	0.002	0.068	< 0.001	0.562
C20:5n3, EPA	0.12	0.12	0.12	0.11	0.01	0.480	0.214	0.670
C22:0	0.07	0.07	0.05	0.06	0.01	0.434	0.866	0.844
C22:1n9	0.15	0.15	0.16	0.13	0.01	0.743	0.204	0.418
C22:2	0.05	0.04	0.04	0.03	0.01	0.082	0.406	0.615
C23:0	0.06	0.07	0.07	0.05	0.01	0.899	0.527	0.178
C24:0	0.04	0.05	0.05	0.04	0.01	0.686	0.892	0.503
C22:6n3, DHA	0.46	0.50	0.45	0.49	0.02	0.337	0.035	0.940
C24:1n9	0.08	0.14	0.11	0.15	0.03	0.467	0.139	0.684
ω-3 fatty acid	0.98	1.16	0.99	1.15	0.03	0.824	< 0.001	0.712
ω-6 fatty acid	7.81	7.58	7.82	7.59	0.03	0.852	< 0.001	0.978
ω-6: ω-3	7.96	6.52	7.93	6.66	0.24	0.824	< 0.001	0.729
ΣSFA	39.38	38.41	39.07	38.78	0.49	0.956	0.224	0.502
ΣUSFA	60.62	61.59	60.93	61.22	0.49	0.956	0.224	0.502
ΣMUFA	51.48	52.51	51.80	52.13	0.48	0.965	0.198	0.497
ΣPUFA	9.14	9.09	9.13	9.09	0.06	0.966	0.421	0.897
MUFA/SFA	1.31	1.37	1.33	1.35	0.02	0.901	0.216	0.487
PUFA/SFA	0.23	0.24	0.23	0.23	0.003	0.901	0.453	0.587
Unknown	0.00	0.00	0.00	0.00	-	-	-	-
Total FA	100.00	100.00	100.00	100.00	-	-	-	-

^1^Abbreviation: SIL: silicate; CP: crude protein; SEM: standard error of means; LA: linoleic acid; ALA: α-Linolenic acid; EPA: eicosapentaenoic acid; DHA: docosahexaenoic acid; SFA: saturated fatty acids; USFA: unsaturated fatty acids; MUFA: monounsaturated fatty acids; PUFA: polyunsaturated fatty acids; FA: fatty acids

The effects of SIL supplementation on the fatty acid profile of loin (lean meat) are shown in Table 7. Significant SIL main effects were observed for C17:0, C18:1c, C18:3n3 (ALA), ω-3 fatty acids, the ω-6:ω-3 ratio, ΣSFA, ΣUSFA, MUFA, and the MUFA/SFA ratio (*P = 0.037*,* 0.021*,* < 0.001*,* < 0.001*,* < 0.001*,* 0.050*,* 0.050*,* 0.026*, and *0.030*, respectively). In contrast, the main effect of dietary protein level and its interaction with SIL supplementation had no significant influence on the fatty acid profile of loin tissue.

**Table 7:** Effects of SIL supplementation at different protein levels in diets on fatty acid profile (loin, lean meat) in finishing pigs^1^

Items	High protein		Low protein		SEM	*P* - value		
	SIL -	SIL +	SIL -	SIL +		CP effect	SIL effect	Interaction
Crude fat	5.41	5.35	5.25	5.37	0.11	0.530	0.810	0.450
C4:0	0.00	0.00	0.00	0.00	-	-	-	-
C6:0	0.03	0.04	0.03	0.04	0.005	0.523	0.254	0.699
C8:0	0.02	0.02	0.01	0.02	0.01	0.602	0.894	0.507
C10:0	0.08	0.08	0.08	0.09	0.003	0.827	0.180	0.220
C11:0	0.00	0.00	0.00	0.00	-	-	-	-
C12:0	0.16	0.16	0.16	0.16	0.01	0.675	0.962	0.956
C13:0	0.00	0.00	0.00	0.00	-	-	-	-
C14:0	1.49	1.43	1.42	1.35	0.06	0.253	0.313	0.960
C14:1	0.02	0.02	0.01	0.02	0.01	0.486	0.794	0.701
C15:0	0.02	0.02	0.03	0.02	0.01	0.529	0.350	0.219
C15:1	0.00	0.00	0.00	0.00	-	-	-	-
C16:0	25.98	24.86	25.58	24.45	0.60	0.756	0.386	0.997
C16:1	3.70	3.59	3.62	3.75	0.19	0.879	0.974	0.513
C17:0	0.20	0.16	0.19	0.15	0.02	0.592	0.037	0.883
C17:1	0.16	0.15	0.17	0.15	0.01	0.433	0.247	0.692
C18:0	11.17	11.00	11.22	11.16	0.02	0.675	0.626	0.810
C18: 1,t	0.01	0.01	0.02	0.01	0.01	0.925	0.913	0.958
C18: 1,c	45.65	47.07	46.26	47.50	0.41	0.317	0.021	0.864
C18:2n6t	0.09	0.09	0.08	0.10	0.003	0.894	0.454	0.414
C18:2n6c, LA	8.63	8.54	8.62	8.42	0.27	0.909	0.494	0.945
C18:3n6	0.00	0.00	0.00	0.00	-	-	-	-
C18:3n3, ALA	0.56	0.81	0.54	0.83	0.04	0.946	< 0.001	0.588
C20:0	0.15	0.16	0.15	0.14	0.01	0.219	0.613	0.320
C20:1	0.72	0.69	0.70	0.64	0.05	0.456	0.345	0.815
C20:2	0.32	0.30	0.29	0.26	0.03	0.306	0.383	0.872
C20:3n6	0.15	0.13	0.14	0.12	0.02	0.519	0.267	0.745
C21:0	0.00	0.00	0.00	0.00	-	-	-	-
C20:3n3	0.41	0.43	0.41	0.42	0.02	0.988	0.517	0.939
C20:4n6	0.05	0.04	0.05	0.04	0.01	0.700	0.173	0.897
C20:5n3, EPA	0.01	0.01	0.01	0.00	0.005	0.646	0.486	0.054
C22:0	0.00	0.00	0.00	0.00	-	-	-	-
C22:1n9	0.02	0.02	0.02	0.01	0.01	0.886	0.477	0.211
C22:2	0.00	0.00	0.00	0.00	-	-	-	-
C23:0	0.12	0.12	0.10	0.11	0.01	0.080	0.755	0.536
C24:0	0.06	0.06	0.06	0.06	0.01	0.282	0.512	0.825
C22:6n3, DHA	0.00	0.00	0.00	0.00	-	-	-	-
C24:1n9	0.00	0.00	0.00	0.00	-	-	-	-
ω-3 fatty acid	0.97	1.25	0.97	1.26	0.04	0.974	< 0.001	0.839
ω-6 fatty acid	8.92	8.80	8.89	8.67	0.28	0.876	0.478	0.959
ω-6: ω-3	9.19	7.05	9.29	6.90	0.36	0.136	< 0.001	0.695
ΣSFA	39.49	38.10	39.04	37.73	0.62	0.518	0.050	0.946
ΣUSFA	60.51	61.90	60.96	62.27	0.56	0.518	0.050	0.946
ΣMUFA	50.29	51.55	50.81	52.09	0.33	0.290	0.026	0.991
ΣPUFA	10.22	10.35	10.15	10.19	0.32	0.820	0.892	0.970
MUFA/SFA	1.28	1.35	1.30	1.38	0.03	0.401	0.030	0.949
PUFA/SFA	0.26	0.27	0.26	0.27	0.01	0.979	0.333	0.940
Unknown	0.00	0.00	0.00	0.00	-	-	-	-
Total FA	100.00	100.00	100.00	100.00	-	-	-	-

^1^Abbreviation: SIL: silicate; CP: crude protein; SEM: standard error of means; LA: linoleic acid; ALA: α-Linolenic acid; EPA: eicosapentaenoic acid; DHA: docosahexaenoic acid; SFA: saturated fatty acids; USFA: unsaturated fatty acids; MUFA: monounsaturated fatty acids; PUFA: polyunsaturated fatty acids; FA: fatty acids

### Fecal microbiota

**Fig. 5:**
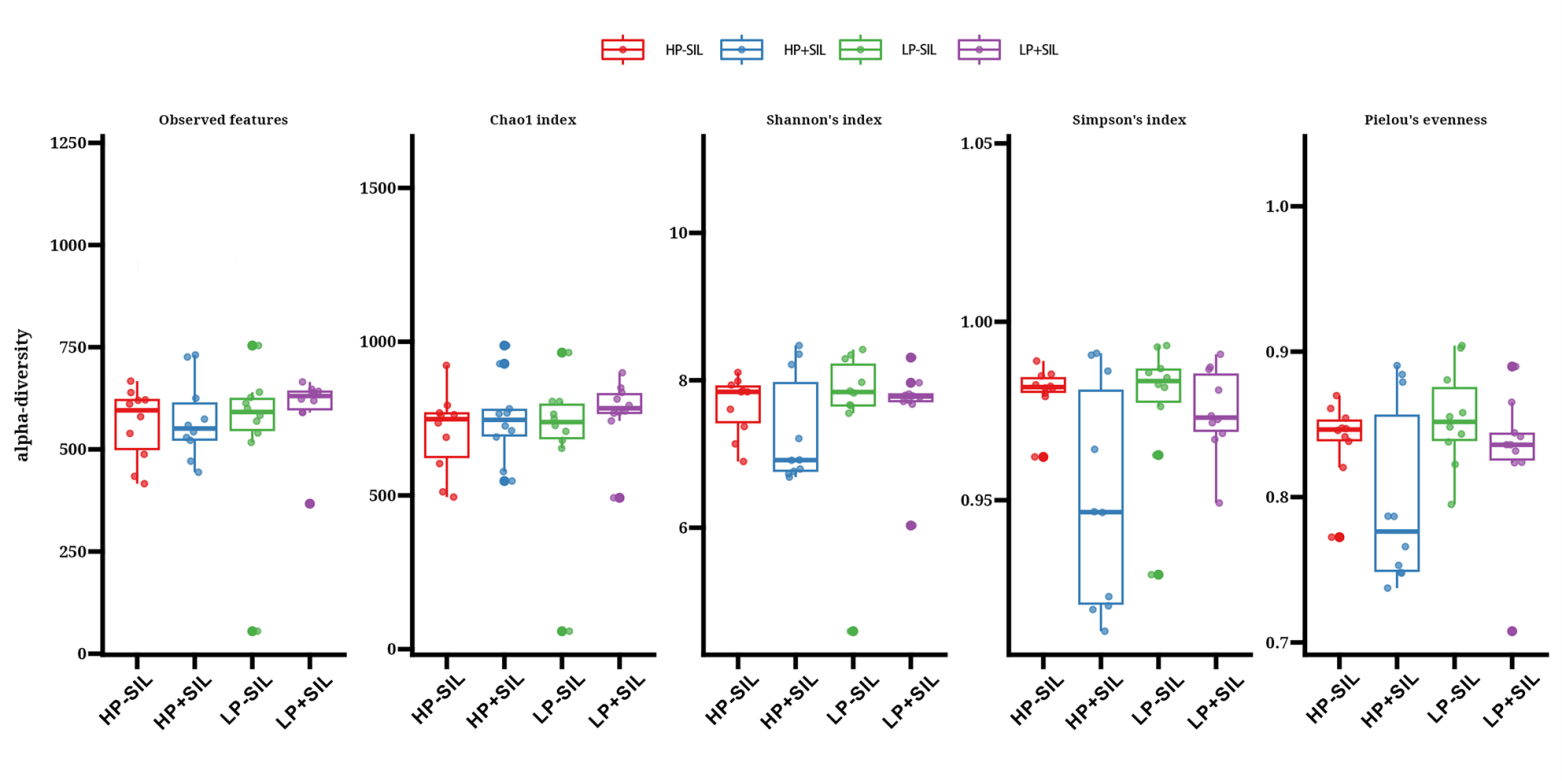
Alpha-diversity of the gut microbiota in finishing pigs fed diets with different protein levels and silicate (SIL) supplementation. Alpha-diversity indices included (**A**) Observed ASVs, (**B**) Chao1, (**C**) Shannon, (**D**) Simpson, and (**E**) Pielou’s evenness. Data were analyzed using a two-way ANOVA with protein level and SIL as fixed effects. SIL showed a significant main effect on Pielou’s evenness (*P < 0.05*), whereas no significant protein main effects or protein level × SIL interactions were detected (*P > 0.05*)

**Fig. 6:**
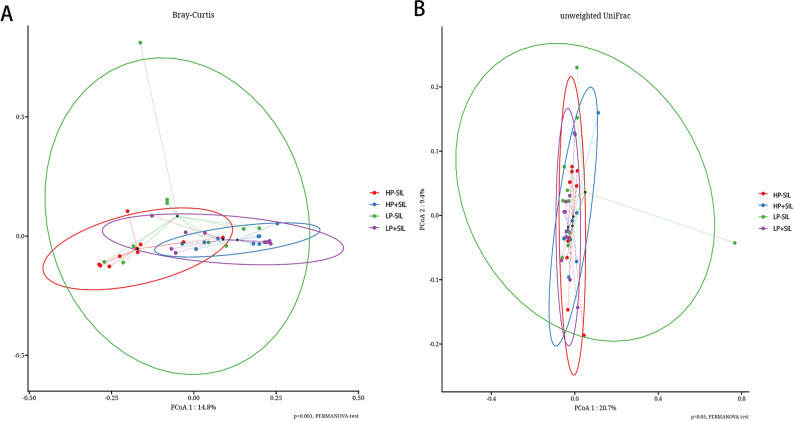
Beta-diversity analysis of gut microbial community structure in finishing pigs fed diets with different protein levels and SIL supplementation. HP, high-protein; LP, low-protein; SIL, silicate. Microbial β-diversity was evaluated based on (**A**) Bray–Curtis dissimilarity and (**B**) unweighted UniFrac distance. Differences in overall microbial community structure among dietary treatments were tested by PERMANOVA

**Fig. 7:**
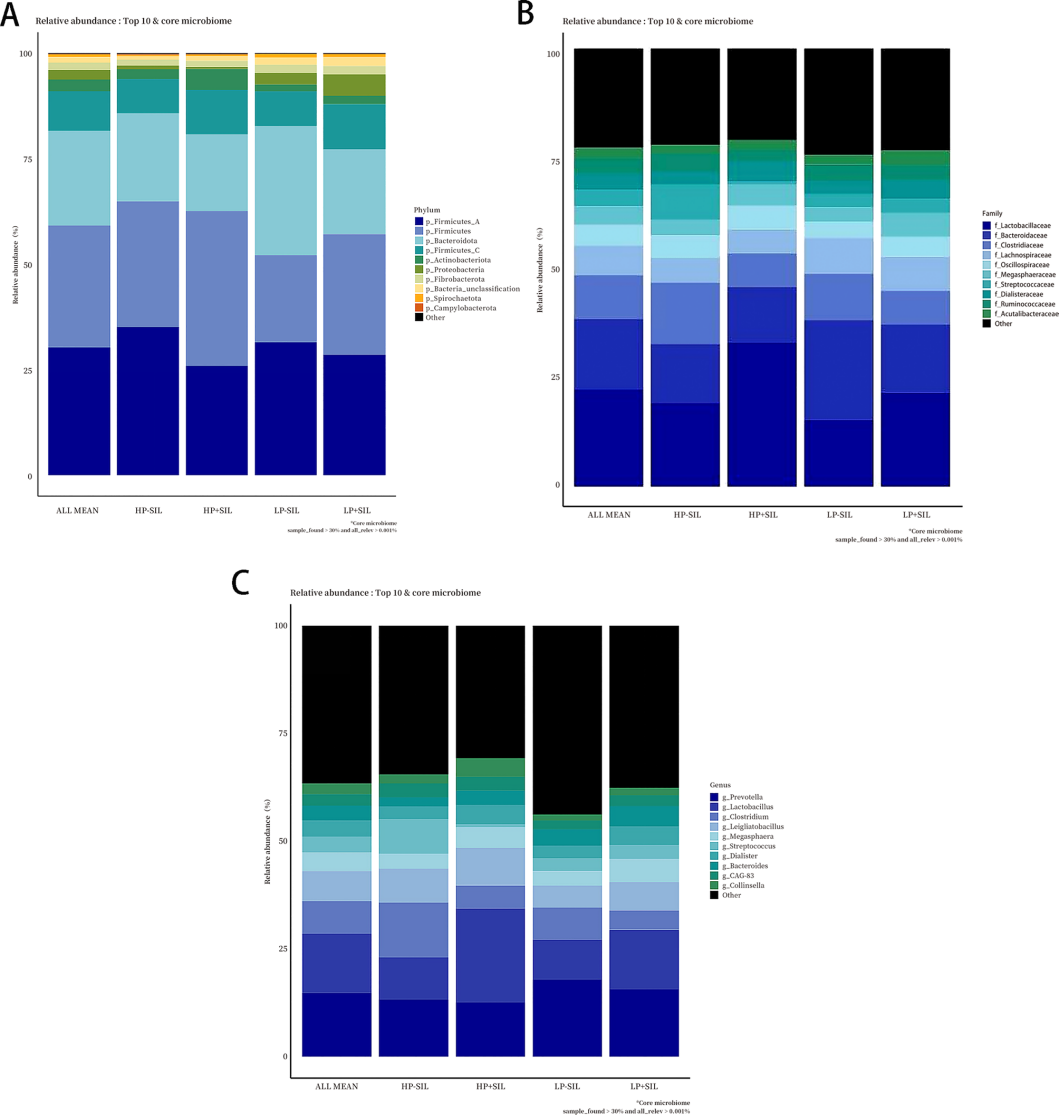
Gut microbiota taxonomic composition of finishing pigs fed diets with different protein levels under SIL supplementation. HP, high-protein; LP, low-protein; SIL, silicate. Relative abundances of gut microbiota are shown at the (**A**) phylum, (**B**) family, and (**C**) genus levels for each dietary treatment group

The effects of SIL supplementation on fecal microbiota composition are presented in Figs. 5, 6 and 7. For α-diversity, two-way ANOVA indicated a significant main effect of dietary SIL supplementation on the Pielou’s evenness index (*P < 0.05*). However, no significant main effects of protein level or protein × SIL interaction were detected for Observed features, Chao1, Shannon, or Simpson indices (*P > 0.05*).

β-diversity analysis based on Bray–Curtis and unweighted UniFrac distance matrices, assessed by permutational multivariate analysis of variance (PERMANOVA), demonstrated that SIL supplementation significantly altered the overall gut microbial community structure (*P < 0.05*). No significant protein × SIL interaction was observed (*P > 0.05*).

At the phylum level, a total of 15 major bacterial phyla were identified. Firmicutes, Bacteroidota, and Proteobacteria were the dominant phyla across all treatment groups, although their relative abundances varied among dietary treatments.

At the family level, approximately 22 bacterial families were detected. Lactobacillaceae, Bacteroidaceae, Clostridiaceae, Lachnospiraceae, and Ruminococcaceae were commonly present as major families across treatments, with their relative abundances differing among dietary conditions.

At the genus level, 31 genera were identified. *Prevotella*, *Lactobacillu*s, *Clostridium*, *Ligilactobacillus*, and *Megasphaera* were the predominant genera across treatment groups, but their relative distributions varied depending on dietary treatment.

**Fig. 8:**
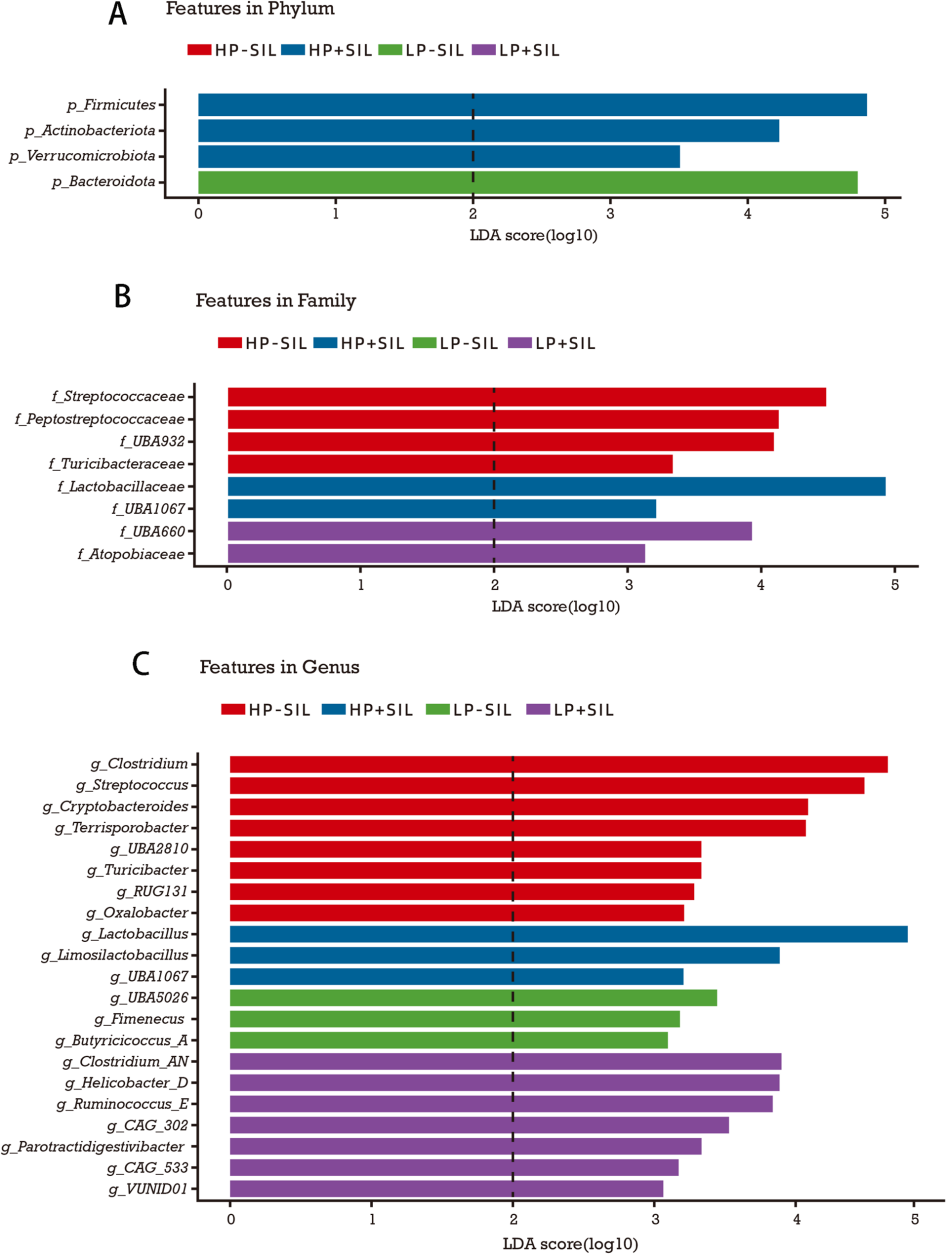
LEfSe analysis of the gut microbiota in finishing pigs fed diets supplemented with SIL at different dietary protein levels. HP, high-protein; LP, low-protein; SIL, silicate. Taxa showing differential enrichment among dietary treatments were identified by LEfSe at the phylum (**A**), family (**B**), and genus (**C**) levels. LEfSe was applied as an exploratory approach, with taxa selected based on an LDA score > 2 and *P* < 0.05 (*n* = 20). Dashed vertical lines indicate the LDA score threshold

LEfSe analysis (Fig. 8) was conducted to explore potential differential microbial taxa among dietary treatments as a complement to conventional factorial statistical analyses. At the phylum level, distinct separations in gut microbial community structure were observed among treatment groups. Firmicutes and Actinobacteriota were more abundant in SIL-supplemented groups, whereas Bacteroidota exhibited higher relative abundance under low-protein (LP) dietary conditions.

At the family and genus levels, LEfSe identified multiple differentially abundant taxa with distinct distribution patterns across dietary combinations. Notably, several lactic acid bacteria–related taxa were enriched in SIL-associated treatments, including the family Lactobacillaceae and the genera Lactobacillus and *Limosilactobacillus*, suggesting a potential association between SIL supplementation and the enrichment of lactic acid bacteria–related groups.

In addition, LEfSe identified several differentially abundant taxa associated with carbohydrate fermentation and SCFA production, such as *Butyricicoccus_A* and *Ruminococcus_E*. It should be noted that LEfSe analysis is an exploratory approach intended to identify potential microbial biomarkers for describing differences in microbial communities among treatment groups, rather than to replace formal factorial statistical analyses.

## Discussion

Growth performance and nutrient digestibility are key indicators of the health status and feed utilization efficiency of growing pigs, directly influencing production performance and economic returns [32]. Previous studies have reported that supplementation of 0.1% SIL in piglet diets can enhance the digestibility of DM, N, and E, thereby improving body weight and ADG [17]. Consistently, the results of the present study demonstrated that dietary supplementation with 0.1% SIL significantly increased DM, N, and E digestibility. This enhancement may be attributed to the stimulatory effect of SIL on pancreatic digestive enzyme secretion or its protective effect on intestinal epithelium, leading to improved absorption and utilization of protein and E [33]. Moreover, ADG was significantly increased throughout the experimental period, while the FCR was significantly reduced during wk 5–10 and across the entire trial. These improvements may be related to the ability of SIL to modify intestinal flow dynamics, prolonging the contact time between nutrients and digestive enzymes, thereby enhancing feed utilization and promoting growth [34]. In contrast, the main effect of protein level was not significant, consistent with previous findings that LP diets supplemented with crystalline amino acids do not impair growth performance in growing pigs but serum creatinine levels showed an increasing trend [35]. Therefore, under appropriate protein intake conditions, dietary SIL supplementation can synergistically enhance nutrient utilization efficiency and promote growth performance in growing pigs.

Fecal characteristics and gas emissions are crucial indicators of intestinal health and environmental sustainability in growing pigs, providing an integrated assessment of digestive efficiency and farm management quality [36]. Previous studies have shown that dietary supplementation with clay minerals such as montmorillonite, zeolite, and kaolinite can reduce the incidence and severity of diarrhea by suppressing harmful bacteria (E coli, Clostridium spp.) and promoting beneficial microorganisms (*Lactobacillus*, *Bifidobacterium*), thereby improving fecal consistency, enhancing nutrient absorption, and decreasing harmful gas emissions [37]. Similarly, SIL analogs such as zeolite-based manure additives have been reported to significantly reduce NH_3_ and H_2_S emissions from pig manure, thereby mitigating environmental pollution in swine production systems [38]. In this study, dietary supplementation with SIL markedly improved fecal characteristics by wk 10, with fecal scores shifting from watery to more formed stools, indicating enhanced stool consistency. This effect may be associated with the moisture-absorbing properties of SIL, while feeding a LP diet can further reduce undigested protein fermentation and optimize the gut microbiota, effectively lowering diarrhea incidence and harmful gas emissions while promoting intestinal health [39]. Additionally, SIL inclusion in the diet significantly reduced emissions of NH₃, H₂S, and CO₂. This may be due to the high cation-exchange capacity of SIL, which allows it to adsorb NH₄⁺, thereby physically immobilizing them and reducing their volatilization [40]. This mechanism further strengthens the role of SIL in mitigating harmful gas emissions. HP diets often lead to excessive amino acid intake; when amino acids exceed the capacity for protein synthesis, the excess N is deaminated and excreted as urea [12]. When urinary urea enters the hindgut and mixes with feces, it is rapidly hydrolyzed by urease-producing microorganisms to form NH₃ and CO₂, which are major contributors to odor and gas emissions in pig manure systems [41]. Thus, reducing dietary protein intake decreases urea production and the N substrate available for NH_3_ generation, lowering emissions at the source. Beyond its physical adsorption effects, SIL may improve nutrient utilization efficiency and reduce gaseous emissions partly through modulation of the structure and function of the intestinal microbiota. Previous studies have shown that SIL can adsorb toxic metabolites and stabilize luminal pH, thereby improving the intestinal physicochemical environment and selectively promoting the proliferation of beneficial bacteria such as *Lactobacillus* [42]. An increased abundance of *Lactobacillus* enhances carbohydrate fermentation efficiency and reduces the availability of undigested substrates for proteolytic bacteria in the hindgut [43]. In addition, short-chain fatty acids (SCFAs) produced by lactic acid bacteria can further decrease intestinal pH, thereby inhibiting the growth of NH_3_-producing microorganisms and improving N retention, which contributes to a reduced N emission load in livestock production systems [44]. Overall, adding SIL to diets with varying protein levels not only improved fecal quality but also significantly reduced harmful gas emissions, supporting a low-carbon dietary approach and highlighting its potential as an environmentally friendly feed additive that promotes both animal welfare and sustainable swine production.

Blood indices reflect the health status, metabolic level, and stress response of growing–finishing pigs, thereby influencing their growth performance and nutrient utilization efficiency [45]. In this study, dietary supplementation with SIL significantly increased WBC counts. This finding suggests that SIL supplementation under HP conditions may be more effective in stimulating systemic immune responses. Similarly, reported that dietary SIL supplementation significantly increased the WBC count in chickens, suggesting an enhanced immune response [46]. SIL minerals may promote intestinal barrier integrity and mucosal immune factor secretion, thereby improving resistance to pathogenic bacteria and toxins and inducing leukocyte proliferation [47]. Previous studies have indicated that LP diets markedly reduce BUN, reflecting optimized protein metabolism [48], When pigs are fed HP diets, excess amino acids must be excreted in the form of urea, thereby leading to elevated BUN levels [49]. Additionally, SIL markedly reduced BUN levels, indicating that under LP dietary conditions, SIL supplementation can further enhance N utilization efficiency. Creatinine, a metabolic product of muscle breakdown excreted by the kidneys, decreased following SIL supplementation, suggesting improved renal function and protein metabolism [50]. A study found that pigs fed SIL based mineral additives such as bentonite and zeolite exhibited significantly lower plasma creatinine and urea levels than the control group [51]. Furthermore, SIL inclusion led to a significant decrease in creatinine levels and a notable increase in blood glucose, suggesting improved protein metabolism and energy status. This effect may be attributed to the adsorption of intestinal toxins and harmful metabolites by SIL minerals, thereby reducing renal excretory load [16]. Additionally, SIL supplementation may improve gut health, enhance nutrient digestibility and absorption, and decrease muscle catabolism, thereby protecting kidney function and optimizing protein utilization [52], while also improving liver function and glycogen metabolism efficiency, resulting in increased circulating glucose [53]. Moreover, SIL may mitigate metabolic disturbances induced by stress (e.g., endotoxemia or heat stress), maintaining insulin and glucose homeostasis at higher levels [54]. Cortisol, a major stress hormone, decreased following SIL supplementation, indicating an anti-stress effect [55]. Clay based SIL minerals such as bentonite and sepiolite can adsorb intestinal toxins and endotoxins and exert pronounced anti-inflammatory effects [45]. Studies have demonstrated that SIL additives can suppress inflammatory cytokine release and alleviate tissue inflammation, thereby attenuating stress responses [56]. Under oxidative or pathogenic stress, such mineral feed additives can modulate signaling pathways such as TLR4/NF-κB, reducing the expression of inflammatory cytokines like IFN-γ and IL-1β, ultimately lowering systemic stress [57].

Meat quality and fatty acid composition are critical determinants of the economic value, nutritional profile, and consumer acceptance of pork, and are key indicators for evaluating dietary regulation and nutritional optimization [5]. In this study, pigs fed diets supplemented with SIL showed significant improvements in WHC, cohesiveness, and springiness, along with a marked reduction in drip loss on day 7, indicating enhanced meat tenderness and juiciness. These improvements are consistent with previous reports showing that enhanced muscle antioxidant capacity stabilizes cell membranes and reduces water loss during processing [58]. Pork belly and loin cuts are of particular consumer interest because their fatty acid composition directly determines flavor, nutritional value, and healthiness [59]. Previous research has shown that SIL supplementation can increase the absorption and synthesis of polyunsaturated fatty acids (PUFAs) while reducing lipid peroxidation, thereby optimizing overall fatty acid distribution [60]. In this study, SIL supplementation significantly affected the fatty acid profile of finishing pigs, while CP levels and their interactions showed no significant effects.

Pork belly, SIL supplementation reduced palmitic acid (C16:0) and total saturated fatty acids (ΣSFA) while slightly increasing arachidic acid (C20:0). It significantly increased oleic acid (C18:1c) and total monounsaturated fatty acids (ΣMUFA), elevating the MUFA/SFA ratio. As the predominant MUFA, oleic acid contributes to pork tenderness and flavor, enhancing both sensory and nutritional quality (Vieira, C., 2021). A higher MUFA/SFA ratio also indicates improved oxidative stability and extended shelf life [61]. Furthermore, SIL supplementation significantly increased α-linolenic acid (C18:3n3, ALA) and arachidonic acid (C20:4n6) contents, as well as total ω-3 fatty acids, while decreasing the ω-6: ω-3 ratio, thereby improving the nutritional profile and reducing off-flavor development [62]. Previously reported that natural SIL such as clinoptilolite may elevate the ω-6/ω-3 ratio, whereas the opposite trend in this study suggests enhanced lipid metabolism and antioxidant stability [63]. This shift toward higher ω-3 fatty acid content and a lower ω-6/ω-3 ratio is widely considered indicative of a healthier lipid profile [64, 65]. Notably, the C22:2 content slightly decreased, possibly reflecting the metabolic conversion and redistribution of long chain polyunsaturated fatty acids during synthesis and deposition. However, this minor fluctuation did not weaken the overall improvement in fatty acid quality [66]. In addition, SIL supplementation significantly elevated total unsaturated fatty acids (ΣUSFA), further confirming enhanced desaturation metabolism and a more favorable fatty acid profile.

Lean pork, In lean belly tissue, SIL supplementation significantly increased heptadecanoic acid (C17:0) while reducing capric acid (C10:0) and palmitic acid (C16:0), suggesting inhibition of short chain and branched chain saturated fatty acid synthesis [67]. A moderate reduction in C16:0 is beneficial for improving fat softness and health value, as excessive saturated fatty acids can cause fat hardening and diminish flavor [68]. Although MUFA content showed no significant changes, PUFA composition shifted markedly SIL supplementation decreased linoleic acid (C18:2n6c, LA) and dihomo-γ-linolenic acid (C20:3n6), while increasing α-linolenic acid (C18:3n3, ALA), eicosatrienoic acid (C20:3n3), arachidonic acid (C20:4n6), and docosahexaenoic acid (C22:6n3, DHA). Consequently, total ω-3 fatty acids increased and ω-6 fatty acids decreased, leading to a significantly reduced ω-6: ω-3 ratio.

Loin tissue, SIL supplementation significantly decreased heptadecanoic acid (C17:0), indicating a consistent reduction in SFA content. This may be due to enhanced desaturation activity or modulation of fatty acid synthase, inhibiting excessive SFA accumulation [69]. Overall, total SFA (ΣSFA) decreased significantly in the SIL group, supporting the hypothesis of improved desaturation metabolism. Additionally, oleic acid (C18:1c) and total MUFA levels increased markedly, elevating the MUFA/SFA ratio, which improves fat stability and meat preservation quality. SIL also significantly increased α-linolenic acid (C18:3n3, ALA) and total ω-3 fatty acids, leading to a lower ω-6: ω-3 ratio. Although total PUFA (ΣPUFA) did not differ significantly, the PUFA/SFA ratio tended to increase, consistent with a more unsaturated fatty acid composition. SIL may regulate fatty acid deposition through multiple mechanisms. SIL acts as a mineral adsorbent, improving gut health, nutrient absorption, and feed utilization efficiency, thereby enhancing lipid synthesis and storage [17]. In addition, SIL significantly altered the intestinal microbiota structure in this study. Gut microbiota and their metabolites, particularly short-chain fatty acids (SCFAs), play critical roles in host lipid metabolism by regulating lipogenic gene expression, fat deposition, and lipid oxidative stability [70–72]. Therefore, SIL may influence host fatty acid biosynthetic pathways by modulating microbial metabolites, selectively promoting the synthesis and deposition of beneficial fatty acids [61].

Overall, SIL supplementation reduced the proportion of saturated fatty acids while significantly modifying ω-3 and ω-6 levels and their ratio across tissues, consistent with a healthier fatty acid balance. These findings suggest that SIL contributes to improved lipid metabolism and meat quality in finishing pigs, demonstrating its potential as a functional feed additive for quality enhancement.

The intestinal microbiota in growing–finishing pigs plays a vital role in regulating nutrient digestion and absorption, metabolic balance, and immune function, thereby significantly affecting growth performance and overall health [73]. In the present study, distinct dietary treatments resulted in significant differences in the overall microbial community structure based on β-diversity analysis, suggesting a potential association between dietary composition and the remodeling of the gut microbiota. These findings indicate that dietary protein level and SIL supplementation, as important nutritional factors, may be involved in shaping the overall compositional patterns of the gut microbial community. At the phylum level, Firmicutes, Bacteroidota, and Proteobacteria were the predominant phyla across all dietary treatment groups, although their relative abundances exhibited certain distributional differences under different dietary conditions. Previous studies have demonstrated that variations in dietary protein levels and mineral-based additives can influence the phylum-level structure of the gut microbiota in pigs [20, 48]. The findings of the present study are generally consistent with these reported trends, suggesting that adjustments in macronutrient composition may affect the gut microbial ecosystem by altering the relative proportions of dominant bacterial phyla. At the family level, several taxa closely associated with gut function, including Lactobacillaceae and Bacteroidaceae, exhibited distinct abundance patterns under different dietary treatments. Members of the Lactobacillaceae family are primarily involved in lactic acid fermentation and play important roles in maintaining gut microbial homeostasis, suppressing the growth of potential pathogens, and improving the intestinal environment. In addition, Lactobacillaceae can participate in the transformation of dietary substrates through fermentation processes, thereby being associated with enhanced nutrient absorption and utilization efficiency [74]. The Bacteroidaceae family is enriched in enzymes capable of degrading complex polysaccharides and plays a crucial role in promoting carbohydrate fermentation and enhancing host energy harvest [75]. At the genus level, dominant genera such as *Prevotella* and *Lactobacillus* exhibited distinct relative abundance patterns among different dietary treatment groups. Previous studies have demonstrated that members of the genus *Prevotella* are capable of efficiently fermenting dietary fiber and starch, leading to the production of SCFAs, including acetate, propionate, and butyrate. These microbial metabolites not only contribute to lowering intestinal pH and inhibiting the growth of pathogenic bacteria, but also act as signaling molecules involved in the regulation of intestinal barrier function and host immune responses [76]. In addition, genera associated with lactic acid fermentation are generally considered to be positively associated with improvements in gut health status and nutrient utilization efficiency [77]. As key metabolic products of the gut microbiota, SCFAs have been well documented to play critical roles in regulating host lipid metabolism, antioxidant capacity, and inflammatory responses [78]. Evidence suggests that SCFAs can influence metabolic pathways related to fatty acid synthesis and β-oxidation, thereby participating in the regulation of lipid deposition and oxidative stability, which may contribute to a more favorable fatty acid profile [79]. Furthermore, metabolic activities associated with lactic acid–producing bacteria may alleviate inflammatory responses and oxidative stress, indirectly improving nutrient utilization efficiency and promoting a more balanced energy allocation within the host [80, 81]. Therefore, the alterations in gut microbial community structure observed in the present study may be partially associated with improvements in fatty acid composition and metabolic phenotypes. LEfSe analysis was applied as an exploratory approach to identify potential differentially enriched microbial biomarkers among dietary treatments.

The relative enrichment of specific taxa under certain dietary combinations suggests that changes in diet composition may influence the functional potential of the gut microbiota. These microbial shifts may be associated with the improvements observed in nutrient digestibility, reductions in N-related gaseous emissions, and changes in immune-related indicators in the present study; however, the underlying mechanisms remain to be elucidated through further functional and mechanistic investigations [82].

Overall, the results of the present study support that a LP dietary strategy combined with SIL supplementation may achieve more favorable nutrient utilization and environmental emission characteristics while maintaining comparable growth performance. Reducing dietary CP levels contributes to lower N excretion and associated gaseous emissions, thereby alleviating metabolic burden in pigs and reducing environmental pressure.

Under LP conditions, the comparable growth performance and higher digestibility of nutrients and E observed with SIL supplementation suggest that this dietary strategy may partially compensate for reduced protein supply by improving digestive efficiency, stabilizing gut function, and modulating microbial fermentation patterns. From an environmental perspective, the combination of an LP diet with SIL supplementation was associated with reduced NH₃ and H₂S emissions, indicating a more efficient N utilization pattern and highlighting its potential as an environmentally friendly feeding strategy in finishing pig production.

## Conclusion

Overall, dietary supplementation with 0.1% SIL represents a more sustainable nutritional strategy. This approach supports growth performance while enhancing nutrient utilization efficiency and reducing gaseous emissions, thereby achieving a favorable balance between production efficiency and environmental responsibility and aligning with the principles of low-carbon and sustainable swine production systems.

## Data Availability

The data presented in this study are available on request from the corresponding author.

## Abbreviations

HP: High protein
LP: Low protein
SIL: Silicate
ADG: Average daily gain
FCR: Feed conversion ratio
DM: Dry matter
CP: Crude Protein
NH₃: Ammonia
H_2_S: Hydrogen Sulfide
CO_2_: Carbon Dioxide
WHC: Water-holding capacity
BUN: Blood urea nitrogen
GHG: Global greenhouse gas
SCFAs: Short chain fatty acids
SiO_2_: Quartz
Na_2_CO_3_: Carbonate
H3PO_4_: Phosphate compounds
BW: Body weight
ADFI: Average daily feed intake
ATTD: Apparent total tract digestibility
Cr_2_O_3_: Chromium oxide
DM: Dry matter
N: Nitrogen
E: Energy
K_3_EDTA: Tipotassium ethylenediaminetetraacetate
RBC: Red blood cell
TPA: Texture profile analysis
PCoA: Principal coordinate analysis
LEfSe: Linear discriminant analysis effect size
LDA: Linear discriminant analysis
ΣUSFA: Total unsaturated fatty acids
